# Implications for public health demands alternatives to inorganic and synthetic flocculants: bioflocculants as important candidates

**DOI:** 10.1002/mbo3.334

**Published:** 2016-02-24

**Authors:** Kunle Okaiyeto, Uchechukwu U. Nwodo, Stanley A. Okoli, Leonard V. Mabinya, Anthony I. Okoh

**Affiliations:** ^1^ SAMRC Microbial Water Quality Monitoring CentreUniversity of Fort HareAlice5700South Africa; ^2^ Applied and Environmental Microbiology Research Group (AEMREG)Department of Biochemistry and MicrobiologyUniversity of Fort HareAlice5700South Africa; ^3^ GenØK – Centre for Biosafety, Science ParkUniversity of TromsøTromsø9291Norway

**Keywords:** Bioflocculants, chemical flocculants, environmental pollution, industrial applications, molecular biology

## Abstract

Chemical flocculants are generally used in drinking water and wastewater treatment due to their efficacy and cost effectiveness. However, the question of their toxicity to human health and environmental pollution has been a major concern. In this article, we review the application of some chemical flocculants utilized in water treatment, and bioflocculants as a potential alternative to these chemical flocculants. To the best of our knowledge, there is no report in the literature that provides an up‐to‐date review of the relevant literature on both chemical flocculants and bioflocculants in one paper. As a result, this review paper comprehensively discussed the various chemical flocculants used in water treatment, including their advantages and disadvantages. It also gave insights into bioflocculants production, challenges, various factors influencing their flocculating efficiency and their industrial applications, as well as future research directions including improvement of bioflocculants yields and flocculating activity, and production of cation‐independent bioflocculants. The molecular biology and synthesis of bioflocculants are also discussed.

## Introduction

Water is one of the key constituents required for extant and thriving of carbon‐based life form (Rani et al. 2013). The bounteous quantity of water on earth is one unique factor that differentiates this planet from others. The importance of water to the survival of life is so crucial that the search for water on other planets has become the key factor to suggest the presence of life (Bhatnagara and Sillanpaa 2010; Rani et al. 2013). Water occupies about 78% of the earth's surface; it is a source of life and energy, nonetheless, millions of people worldwide lack access to safe water for drinking purposes and human utilization (Rout and Sharma 2011). The quality of water consumed by people in a particular community can be taken as a key indicator of the quality of the individual's life within that environment. Water is exceptionally important for domestic, agricultural, industrial, and environmental purposes (Kumar et al. 2005). However, impurities in the water reduce its effective usage as the negative impact of water pollution has minacious effects on man and his environment.

Water pollution is one of the most challenging environmental issues and has become a global impediment to a good quality of life for many communities. Unplanned urbanization and expeditious growths in populations have immensely contributed to the parlous state of water pollution and the prevailing unhealthy environment (Prasertsan et al. 2006). The major source of water pollution is the discharge of domestic and agricultural wastes, and untreated sanitary and toxic industrial effluents (Li et al. 2013). The presence of pollutants in water bodies can be pernicious to aquatic life as well as render it unsuitable as potable water sources for domestic usage. The pollution of the freshwater environment has a life‐threatening effect on man's healthy living (Yang et al. 2012).

According to WHO/UNICEF (2000), about 70–80% of all illnesses in developing countries are linked to the consumption of contaminated water especially among vulnerable population groups (Bhatnagara and Sillanpaa 2010). Pollutants from wastewaters, when discharged into natural water bodies, becomes toxic to aquatic life and render the waters unfit for consumption. The result of this is the alarming increase in waterborne diseases, as well as an increase in the demand for safe water for both municipal and industrial purposes. Much attention has been focused on water treatment, thus making it imperative to appraise water quality on a perpetual basis (Yang et al. 2012). In order to provide these services adequately to meet consumers’ demands, it is incumbent upon governments and societies at large to develop, among other things, appropriate scientific strategies in wastewater treatment technology that are not only environmentally friendly, but also cost‐effective. Of utmost importance is the development of a novel strategy in the wastewater treatment technology to encompass a stricter environmental policy on the quality of the final effluents released into water bodies (Wong et al. 2006). Many countries have inaugurated several stringent regulations with respect to the presence of contaminants in water, to ensure proper treatment of domestic and agricultural wastewater as well as industrial effluents prior to their discharge into different waterbodies (Bhatnagara and Sillanpaa 2010; Li et al. 2013).

## Flocculation Process in Water Treatment

In most water treatment plants, water from the reservoir passes through the first compartment into which flocculants are added. The water then moves to the sedimentation tank where the flocculation process occurs and suspended particles settle at the bottom of the tank. The clarified water from this stage goes through a filtration process prior to being disinfected for distribution to end users. The main reaction stage where natural organic matter and other contaminants are removed is the flocculation stage (Jarvis et al. 2012; Rong et al. 2013). Flocculation is a process whereby colloids, cells, and suspended solids are removed from the suspension. The solids simply look like flocs or flakes as a consequence of aggregation (Bhunia et al. 2012). Flocculants are substances that are used in the separation of solid–liquid by the process of flocculation in various industrial processes (Hu et al. 2006), they could be of natural or synthetic origin. The larger the size of the particle, the faster the sedimentation rate, resulting in an efficient and rapid flocculation process that produces a clearer upper phase (Lachhwani 2005).

Flocculants are commonly used in the various industrial processes, for example, drinking water purification, wastewater treatment, and downstream processes in the fermentation industries (Shih et al. 2001). Shih and Van (2001) found that flocculation could be exploited as a substitute for filtration and centrifugation in the separation of microbial cells from broth in food, beverage, and pharmaceutical industries. In addition, Deng et al. (2003) observed that flocculation is an effective technique that is commonly used in wastewater treatment for removing various suspended particles as well as metal ions.

According to the flocculation mechanism proposed by Wang et al. (2011), for the flocculants to adsorb onto the surface of the suspended particles, it must not only be in close proximity to the suspended particles, but must also exert a strong enough attractive force to overcome the electrostatic repulsion force. In addition, an efficient and rapid flocculation process depends, among other things, on the suspended particle size, which implies that the larger the size the faster the settling rate (Lee et al. 2012). The choice of flocculant has a major influence on the performance of the flocculation process, the strength of the aggregated particles and the number and strength of the bonds formed as a result of flocculation (Zhang et al. 2014). For example, the flocculation efficiency and strength of the bonds of polyelectrolytes is greater than that of ferric chloride.

However, despite the high efficiency of the flocculation process in water treatment, the major disadvantage of flocculation is that it generates small flocs when flocculation occurs at low temperatures or generates fragile flocs that can disperse on the application of physical force (Lee et al. 2014). Consequently, it is crucial to surmount these problems and improve the flocculation processes in order to optimize its effective utilization.

## Classifications of Flocculants

**Figure 1:**
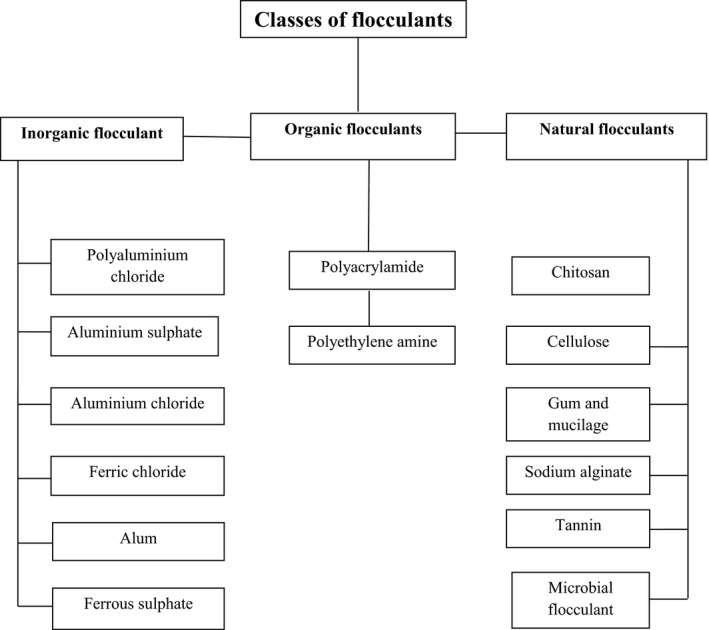
Classes of flocculants.

Flocculants have been used for various wastewater treatments, drinking water purification, and dredging/downstream processes in a variety of industrial fields (Salehizadeh and Shojaosadati 2001). Flocculants are generally categorized as inorganic flocculants, organic flocculants, and naturally occurring flocculants (Fig. 1).

### Inorganic flocculants

Inorganic flocculants include, among others, alum, polyaluminum chloride (PAC), aluminum chloride, aluminum sulfate, ferric chloride, and ferrous sulfates. Since most of the suspended particles in wastewater usually exhibit a negative charge (Lee et al. 2014), the salt of these metals will be ionized when they are added to wastewater to form cationic charges which can bind to the negatively charged suspended particles. This interaction leads to a reduction in surface charge and the formation of microfloc which in turn aggregates to form larger flocs that can easily settle out of solution (Suopajarvi et al. 2013). Among these inorganic flocculants, PACs are widely used in drinking water and wastewater treatment. However, they are very sensitive to pH, inefficient at low temperatures, limited to only a few disperse systems, and large amounts are required for effective flocculation, thus generating a large volume of sludge which is challenging in wastewater treatment plant systems (Wei et al. 2003; Bratby 2006; Sharma et al. 2006). Consequently, it is essential to instigate effectual technologies that will be a logical and effective in the recycling of excess sludge. Furthermore, several studies have reported that PAC contains aluminum which could contaminate drinking water, and lead to serious health problems for consumers (Banks et al. 2006).

Recently, inorganic polymeric flocculants such as ferric polysilicates have been discovered, although they have a lower molecular weight and flocculating efficiency compared to organic polymeric flocculants (Shi and Tang 2006; Moussas and Zouboulis 2008). In addition, regardless of the flocculating capability of modified ferric polysilicates over ferric sulfate, the addition of polysilicic acid, which is negatively charged, will upset the destabilization ability of the modified flocculant, since the positive charges on iron species will be compromised (Moussas and Zouboulis 2009). Subsequently, it is vital to subdue these aforesaid challenges in order to increase flocculating efficiency.

Composite inorganic–organic coagulants such as poly‐dimethyl‐diallyl‐ammonium chloride (PDMDAAC) are normally made by grafting a cationic inorganic coagulant on organic polymers in order to derive a holistic flocculating efficiency from the attributes of both molecules (Moussas and Zouboulis 2009). In recent times, the idea of utilizing composite flocculants in wastewater treatment has attracted more attention and several workers have reported on them (Shi and Tang 2006; Wang et al. 2007a,b; Gao et al. 2008). For instance, Gao et al. (2008) observed that, on treating kaolin suspension or dye solution with polyferric chloride poly‐dimethyl‐diallyl‐ammonium chloride (PFC‐PDMDAAC), a higher flocculating efficiency than that of the individual reactive components (PFC and PDMDAAC) was observed. Furthermore, the addition of this composite flocculant to dye solutions for textile industries generated a high flocculating efficiently. The flocculating competence depends on the percentage of organic polymer used, since PFC‐PDMDAAC carries a higher cationic charge when compared to PFC (Gao et al. 2007; Wang et al. 2007a,b). Nonetheless, the application of these composite flocculants is narrow because they can only be efficient in treating specific samples as a result of the high cationic charge they possess (Moussas and Zouboulis 2008).

### Organic flocculants

Organic flocculants are conventionally utilized in different industrial processes as flocculation agents (Kang et al. 2007). They include polyacrylamide (PAA), polyethylene amine, and poly(diallyl dimethyl ammonium chloride) (DADMAC) (Singh et al. 2000). Moussas and Zouboulis (2009) documented that acrylamide derivatives are major groups of organic synthetic polymers that are widely used as flocculating agents because of their effectiveness and cost efficiency. According to Suopajarvi et al. (2013), these organic polymers are commonly derived from oil‐based or nonrenewable raw materials. They usually have a high molecular weight and possess numerous charges (polyelectrolytes) in their molecular chain which enhance their flocculating effectiveness (Lee et al. 2014). The amount of sludge generated in wastewater treatment can be reduced by using synthetic polymers such as PAA, which are also not sensitive to pH (Huang et al. 2014).

Furthermore, the use of nonionic organic polymers such as PAA can overcome some of the previously mentioned problems encountered with inorganic flocculants. A combination of PAA and polyferric sulfate (PFS) should give an increased flocculating efficiency since PAA, on its own, is a good flocculating agent. Combining PAA with PFS should give rise to a higher molecular weight polymer, thus enhancing its flocculating efficiency when compared to an inorganic flocculants. In addition, PAA is a nonionic polymer, which does not contribute additional charges to the flocculation process and thus has no effect on the destabilization capacity of the inorganic cationic coagulant (Moussas and Zouboulis 2009).

Acrylamide is crystalline in nature. It is a moderately stable monomer that is extremely soluble in water and many organic solvents (Wong et al. 2006). It is a polyfunctional molecule that comprises both a vinylic carbon–carbon double bond and an amide group with a deficient double bond that is prone to a broad scope of chemical reactions (Girma et al. 2005). However, the monomers of the PAA are not easily biodegradable, thereby constituting an environmental nuisance. Besides, these monomers have been reported to have both neurotoxic and carcinogenic properties (Li et al. 2008). Consequently, these demerits associated with them have discouraged their use in most countries.

## Naturally Occurring Flocculants

### Chitosan

Chitosan is a partially deacetylated polymer derived from alkaline deacetylation of chitin, a biopolymer obtained from shellfish sources (Lee et al. 2014). It is a cationic polysaccharide which serves as a synthetic polymeric flocculant that can be applied in the coagulation of suspended particles in the water treatment process because of its safety, noncorrosiveness, and biodegradability (Defang et al. 2008). It is a linear hydrophilic amino polysaccharide which has several amino groups (–NH_2_) and hydroxyl groups (–OH) on its structure. These –OH and –NH_2_ groups have lone‐pair electrons that can donate an electron pair to empty d‐trajectories of metal ions, thereby chelating into a complex compound (–N–M–O–).

Chitosan is insoluble in water as well as in concentrated organic solvents. However, it is soluble in dilute organic solvents (Szyguła et al. 2009). At low pH, chitosan exists as a soluble cationic polymer possessing a high charge density (Rinaudo 2006). When chitosan dissolves in acids, it produces protonated amine groups that can remove various unwanted metal ions such as Ag^+^, Pb^2+^, Ca^2+^, Cu^2+^, Al^3+^, Zn^2+^, Cr^2+^, Hg^2+^, and Cd^2+^ present in the wastewater through electrostatic attraction (Defang et al. 2008).

Jaafari et al. (2004) observed that chitosan has a strong electrostatic and adsorption power owing to the fact that the amino groups (−NH_2_) in the molecular chain could be protonated with H^+^ in water to form cationic NH_3_^+^. Consequently, it can flocculate smaller particles into bigger flocs which can easily be precipitated out of solution. Chitosan has been reported to be effective in the removal of the chemical oxygen demand (COD) of water contaminated with organic solutes and suspended solid substances (SS) in water treatment (Bolto 1995; Ishii et al. 1995). It has numerous advantages over the traditional chemical flocculants that are widely used in water treatment. These advantages include a lesser dosage requirement, a faster sediment ingrate, a high COD reduction capability, suspended solids (SS), and metal ions. In addition, it is used to reduce the production of the large quantity of sludge usually generated by inorganic flocculants and it does not cause any secondary pollution. Due to the high density of chitosan, it increases the floc size, which in turn increases the floc settling rate and decreases the sedimentation period (Renault et al. 2009). Although chitosan is effective in water treatment, it is expensive; therefore, its usage might inflate overall treatment costs.

### Sodium alginate

Sodium alginate is a linear water‐soluble anionic polymer that is derived from the sodium salt of alginic acid and has a molecular weight of about 500,000 (Wu et al. 2012). Recently, Wu et al. (2012) examined its flocculating capability in combination with aluminum sulfate as the coagulant in the treatment of industrial textile wastewater contaminated with synthetic dyes, and found that it exhibited strong flocculating rates of about 90% and 80% for color removal and COD reduction, respectively.

### Tannin

Tannin is an anionic polymer that has been reported to be a safer flocculant which can conveniently be used as a substitute for the commonly used polymers in water treatment due to its biodegradability and safety to humans and the environment Ozacar and Sengıl (2000). Tannin is obtained from the secondary metabolites of vegetables such as fruits, leaves, and others (Beltran‐Heredia and Sanchez‐Martin 2009). Lately, several researchers have experimentally confirmed the flocculating capability of tannin in eliminating suspended and colloidal particles found in drinking water after treatment, as well as in the removal of suspended particles from synthetic raw water and the removal of dyes, pigments, and inks from ink‐containing wastewater (Ozacar and Engil 2003; Roussy et al. 2005). In these studies, and because tannin is anionic in nature, a coagulant such as aluminum sulfate was added in order to stabilize the negatively charged colloidal particles prior to the addition of tannin (Lee et al. 2014).

### Cellulose

Cellulose is one of the most abundant natural polysaccharides obtained from agricultural wastes (Lee et al. 2014). In recent years, cellulose has been the subject of research because of its numerous industrial applications (Das et al. 2012). Anionic sodium carboxymethyl cellulose (CMCNa) is a typical example of flocculant prepared from cellulose, which has been shown to be ecofriendly and has been used, complexed to aluminum sulfate, as a coagulant for the removal of turbidity in drinking water (Khiari et al. 2010). Suopajarvi et al. (2013) reported that anionized dicarboxylic acid nanocellulose (DCC) flocculant derived from cellulose had a strong flocculating property in the presence of ferric sulfate in municipal wastewater treatment.

### Exopolymeric substances

Lately, demand for biopolymers for diverse industrial applications has resulted in an interest in the production of exopolysaccharides (EPS). They are usually complex long‐chain, high‐molecular‐weight mixtures of polymers comprising branched repeating units of sugars or sugar derivatives such as fructose, galactose, glucose, and mannose which are produced and released during the growth of microorganisms (Ismail and Nampoothiri 2010; Sheng et al. 2010).

### Bioflocculant

Among the numerous exopolymeric substances (EPSs) reported in the literature, those that have flocculating properties are particularly interested in the field of bioflocculation and this suggests their candidature for application in water treatment and other industrial processes. Owing to the limitations of these inorganic and organic flocculants, biopolymers produced by microorganisms during growth, called bioflocculants, have gained huge scientific attention because they are biodegradable, produce no secondary pollution, and their degraded intermediates are safe for humans and their immediate environment (Buthelezi et al. 2010; Mabinya et al. 2012). They are pH independent and generate less sludge, which is easily degraded by microorganisms.

However, the major limiting factors that hinder their large‐scale production and industrial application are low flocculating efficiency, low yields, and high cost of production (He et al. 2010; Zhao et al. 2012). Consequently, it has become imperative to identify and screen new bioflocculant‐producing microorganisms and investigate strategies for the optimization of fermentation conditions to improve on bioflocculant yields or on utilizing microbes in a consortium to increase bioflocculant yields (Yang et al. 2007; Okaiyeto et al. 2013).

Abdel‐Aziz et al. (2011) observed that certain enzymes that subsist in clusters regulate the utilization of nutrients in the medium by microorganisms to produce polymers that have a high molecular weight, and which when released, can exist in the medium or form a capsule on the surface of the bacteria. EPSs are often called exopolysaccharides due to their location. This is to differentiate them from other forms of polysaccharides that may be found within the cell (Nwodo et al. 2012). They usually exist as a layer on the surface of the organism, thereby protecting the cell wall against adverse environmental conditions such as high osmotic pressure, oxygen tension, temperature, and toxic compounds. Furthermore, they may contribute to the uptake of metal ions as well as prevent dehydration under certain environmental conditions (Nichols et al. 2005). The capsular polysaccharides are normally extremely immunogenic, and may have changed their unusual diversity as a way of evading antibody responses as is the case in their use in the development/design of vaccines. In addition, they play a vital function in the adhesion and penetration of the host (Morris and Harding 2009). According to Wingender et al. (1999), the release of enzymes by microorganisms into their external environment forms the center of contact between the exogenous substrate and the cells. EPSs are the most important constituent of biological aggregates responsible for the degradation of organic matter in wastewater treatment, which also includes biofilms and activated sludge (Martín‐Cereceda et al. 2001).

EPSs promote the development of bioflocs by amending the relationship among microbial aggregates, different bacterial strains, as well as both organic and inorganic particles. In addition, their fundamental role is to hold the cells firmly together (Li and Yang 2007; Sheng et al. 2010). They can be classified into two types: sheath and slime. Sheath EPSs are tightly bounded to the cell wall, and are commonly called tightly bound exopolysaccharides (TB‐EPS). Slime EPSs have no directed contact with the cell. They loosely/weakly attach to the cell wall and they are usually called loosely bound exopolysaccharides (LB‐EPS). Centrifugation is the basis for the separation of these two fractions (Anna et al. 2006; Sheng et al. 2010). EPSs are usually complex in nature with heterogeneous substances, whose constituents and location can be contingent on several metabolic processes such as active secretion, changes in a growth phase, cell breakage due to cell death, release of cell surface macromolecules (outer membrane proteins and lipopolysaccharides), and their interaction with the immediate environment (Cristina et al. 2011).

Recently, several microorganisms such as algae, bacteria, actinomycetes, and fungi have been implicated in the production of bioflocculants (Gong et al. 2008; Xia et al. 2008; Ugbenyen et al. 2012; Ntsaluba et al. 2013; Cosa and Okoh 2014; Nwodo et al. 2014; Okaiyeto et al. 2014). Although a number of reports are available for EPSs produced by different bacteria found in different habitats, the marine environment, which supports a rich biodiversity of bacteria, remains largely unexplored (Kumari et al. 2014). Li et al. (2008) observed that the majority of the bioflocculants documented in the literature are exopolysaccharides (EPS) which are secreted by microorganisms which were isolated either from the soil or wastewater sludge. Table 1 depicts the advantages and disadvantages of inorganic, organic, and naturally occurring flocculants.

**Table 1:** Advantages and disadvantages of inorganic, organic, and naturally occurring flocculants

Flocculant	Advantage	Disadvantage
Inorganic flocculant	They are cost‐effective and easily available in the market. They have high flocculating efficiency. Their flocculation mechanisms are well established. No production process is required and hence, the problem of skilled personnel is eliminated.	Large amount of inorganic flocculant is required for the flocculation process and aluminum salts produce a lot of sludge whose disposal itself is another problem. Highly sensitive to pH. These flocculants are applicable only to a few disperse systems and do not work for others. They do not coagulate very fine particles. They are inefficient in cold water especially polyaluminum chloride (PAC). Aluminum salts have neurotoxicity effect. Ferrite flocculants lead to excess iron, causing unpleasant metallic taste, odor, color corrosion, foaming, or staining.
Organic flocculant	They have high flocculating efficiency. They are cost‐effective compared to bioflocculant. The molecular weight, molecular weight distribution, nature and percentage of ionic charge, and the very structure of the polymer itself can be varied. They are not sensitive to pH. They can coagulate very fine particles. They are effective in both cold and warm water. They generate lesser sludge compared to PAC.	Nonbiodegradable and hence toxic to the environment. The monomers of polyacrylamide are carcinogenic and neurotoxic. They constitute environmental pollution.
Naturally occurring flocculant	They are harmless. They are biodegradable. They lack secondary pollution. They are cheap except bioflocculant that the production cost is high. They have molecular weight with a definite chain length and molecular constitution. The functional groups can be derivatized to get effective flocculants. They generated lesser sludge. They are biocompatible. They have benign nature. They are not sensitive to pH. They can coagulate very fine particles. They are effective in both cold and warm water. They generate lesser sludge compared to PAC.	Natural polymers have shorter shelf life because its active components will biodegrade with time. Low flocculating activity compared to both inorganic and organic flocculants. The flocs tend to loose stability and strength with time because of their biodegradability. Their flocculation mechanisms are not well understood in details. Large dosage requirement for an effective flocculating efficiency especially bioflocculant. Production cost for bioflocculant is high and low yield has been the major problem.

## Growth Phases During the Process of Bioflocculant Production

The typical bacterial growth curve involves four phases which are lag, log/exponential, stationary, and death phase. The kind of the nutrients in the media is a predetermined factor which play significant role in all the phases of bacteria growth with respect to time. The production of bioflocculant is either growth associated, growth synonymous, or growth independent (Barker and Strucker 1999; More et al. 2014).

At the lag phase, the rate of bacteria multiplication is usually low as the bacteria adapt to the new environment. Although the degree of adaption and the duration vary with different microorganisms as some are fast grower and others are slow grower. Generally in most studies documented in the literature, the flocculating activity of the bioflocculants was usually low at the lag phase of bacteria growth.

As the incubation period increases, the number of cells increases in an exponential rate in the logarithmic phase because of the abundant of nutrient (Nwodo and Okoh 2013). Mostly, the flocculating activity of bioflocculant is usually concomitant to the cell growth at logarithmic growth phase. Some researchers have reported the highest bioflocculant produced from bacterial strains at logarithmic growth phase. For example, the maximum flocculant production of *Alcaligenes latus* was achieved at the middle and late stage of the logarithmic growth phase (2–3 days), and flocculating activity began to decrease during the late stationary phase due to the activity of deflocculation enzymes (Kurane and Nohata 1991).

As the nutrients got depleted from the culture, the oxygen level available for the microorganisms become reduced, and the toxic waste products of metabolic activity become increased. These activities drastically affect the growth of the bacteria thereby reducing the number of viable cells which might be due to the accumulation of dead cells and other metabolic waste products (Li et al. 2009) or production of bioflocculant‐degrading enzymes by the microorganism or accumulation of the toxic metabolic waste products affecting the secondary metabolite that was produced. The bacteria is said to be in the stationary phase of growth, and the rate of cells multiplication equals to the rate at the cells are dying and hence, the flocculating activity of the produced bioflocculant remains stationary. In accordance with Salehizadeh and Yan (2014) reports, bioflocculant production reached maximum flocculating activity both in the late logarithmic growth phase and early stationary phase. At this growth phase, the bioflocculant produced inside the medium can also be degraded by the bacteria to serve as source of carbon and energy (More et al. 2014).

Many researchers have documented that the production of bioflocculant was associated with cell growth and reach its maximum flocculating activity in the early stationary phase of growth (Xia et al. 2008). For example, the production of bioflocculant by *Aspergillus flavus* was growth associated with the highest flocculating activity of 87.2% after 60 h at the early stationary phase (Aljuboori et al. 2013). A similar finding was observed with the bioflocculant MBF‐6 produced by *Klebsiella pneumoniae* YZ‐6 in which the production was parallel with the cell growth, and the highest flocculating activity of 91.5% was attained at the early stationary phase of growth at 60 h. Yang et al. (2012) reported that the bioflocculant produced by *Klebsiella* sp. reached its maximum flocculation rate of 86.5% at 60 h. Gomaa et al. (2012) found that the bioflocculant produced by *Pseudomonas aeruginosa* reached peak flocculation of 62.25% after 72 h of cultivation time. This finding is in agreement with the reports of Raza et al. (2012) where maximum bioflocculant produced by *Pseudomonas* sp. was attained in the early stationary phase after 72 h of fermentation. The productions of these bioflocculants were almost in parallel with the cell growth and the flocculating activities increased with increase in fermentation period which indicated that the bioflocculants production were associated with cell growth. Subsequently, the flocculating activity decreases due to the presence of bioflocculant‐degrading enzyme. The same explanation have used to describe the phenomenon that occurred in the production of bioflocculants by other pure strains (Lu et al. 2005; Gong et al. 2008; Li et al. 2009; Elkady et al. 2011; Okaiyeto et al. 2015a).

When the nutrient has been completely utilized, the rates at which the cells die are usually higher than rate at which they multiply and this consequently reduces the cell population drastically. The cells lysis and release the intracellular materials to the surrounding medium. For example, in the case of the bioflocculant produced by *Corynebacterium daeguense*, the production was not associated with the cell growth but with cell autolysis as flocculating activity increased sharply (over 90%) during the death phase. This implies that it is an intracellular bioflocculant that was produced as a result of the release of intracellular materials of the bacteria (Liu et al. 2010a).

## Factors Affecting the Production of Bioflocculants

According to the available literature, the production of microbial flocculants is highly influenced by the culture medium composition and several other physicochemical parameters (Sheng et al. 2006; Wang et al. 2010; Fang et al. 2013). In addition to these findings, He et al. (2004) documented that the production of bioflocculants is influenced by numerous factors that include the media constituents as well as growth conditions. The impacts of the nutritional constituents of the production of bioflocculants have been widely investigated (Abdel‐Aziz et al. 2011). The impact of the main factors, such as carbon source, culture time, metal ion, initial pH of the production medium, shaking speed, nitrogen source, ionic strength, incubation temperature, and inoculum size greatly influence bioflocculant production (He et al. 2004). Commonly, an appropriate medium for bioflocculant production consist of glucose or fructose as the sole carbon source. However, lactose and yeast extract have been used as the carbon and nitrogen sources, respectively (Kanmani et al. 2011). In addition, small amounts of phosphates and ions are essential (Fujita et al. 2000).

## Effect of Carbon and Nitrogen Sources on Bioflocculant Production

Carbon sources play a substantial role in enhancing the secretion of bioflocculants by microorganisms (Goo et al. 2013). Salehizadeh and Yan (2014) have referred to a number of studies that have acknowledged the significance of carbon and nitrogen sources in the production of bioflocculants. Lee et al. (2001) reported that *Bacillus licheniformis* X14 favored ethanol, sucrose, and starch as appropriate carbon sources for the secretion of ZS‐7 bioflocculant, whereas ammonium chloride was preferred as a nitrogen source of choice.

In the case of another study carried out by Sheng et al. (2006) on *Klebsiella* sp. in the production of bioflocculant, it was documented that maltose and urea were the preferred carbon and nitrogen sources, respectively. Cosa et al. (2013a) observed that sodium carbonate and tryptone were most favorable for bioflocculant production by *Oceanobacillus* sp. Pinky, while tryptone was a preferred organic nitrogen source for bioflocculant production by *Chryseobacterium daeguense* W6 (Liu et al. 2010a). Gong et al. (2003) found that sucrose, maltose, xylitol, lactose, and glucose are all suitable substrates for the production of bioflocculant by *Paenibacillus polymyxa* BY‐28. He et al. (2004) documented a novel polygalacturonic acid bioflocculant REA‐11 produced by *Corynebacterium glutamicum* from sucrose as the carbon source and complex nitrogen sources comprising urea and corn steep liquor. The ability of the microorganism to utilize sucrose as a carbon source for the production of bioflocculant points to the possibility of using molasses in large‐scale industrial bioflocculant production. Cosa et al. (2013b) found that glucose was the preferred carbon source among other sources investigated for bioflocculant production by *Virgibacillus* sp., while Deng et al. (2005) showed sucrose, corn starch, glycerol, and glucose as appropriate substrates for bioflocculant production by *Apergillus parasiticus*, exhibiting a high flocculating activity above 80% at 72 h of fermentation. The production of bioflocculants was optimal when maltose was utilized as a carbon source in the medium for the cultivation of *Solibacillus silvestris* W01 (Wan et al. 2013). For example, soluble starch was the carbon source that supported optimal bioflocculant production by *Sorangium cellulosum*, while the inclusion of glucose as a supplement at 3 g/L entirely repressed cell growth and production of the bioflocculant (Zhang et al. 2002).

In another study reported by Shih et al. (2001), glucose, fructose, and lactose were not suitable for bioflocculant production by *B. licheniformis*, whereas the concurrent presence of multiple carbon sources (glycerol, citric acid, and glutamic acid) in the cultivation medium improved cell growth and the production of bioflocculants. Liu and Chen (2010) recorded an increase in bioflocculant production by *Penicillium* sp. HHE‐P7 in the medium containing glucose and yeast extract. Glucose was the most favorable carbon source for bioflocculant secretion (95% flocculating activity) by microorganisms, but the high cost of glucose inflates the production cost. However, when molasses was substituted for glucose, flocculating activity for kaolin suspension was more than 90%, a clear indication of cost saving when cheaper substrates are used.

Substantial evidence has shown that some bacterial strains can utilize either organic nitrogen source, inorganic nitrogen, or their combination sources produce bioflocculant (Deng et al. 2005; Gong et al. 2008; Xia et al. 2008). For instance, Deng et al. (2005) reported that peptone combined with sodium nitrate was the most suitable nitrogen source for *A. parasiticus* for bioflocculant production. On the other hand, when combined with (NH_4_)_2_SO_4_, no bioflocculant was produced. Nevertheless, organic nitrogen sources improved bioflocculant production in some microorganisms. For example, beef extract and urea used together as a nitrogen source were more favorable for the production of bioflocculant by the S‐14 strain. Xia et al. (2008) found that strain TJ‐1 was able to effectively utilize peptone, yeast, and beef extract as a nitrogen source, but peptone alone (organic nitrogen source) was the most cost‐effective with high bioflocculant production. Cosa et al. (2013b) reported that a complex nitrogen source consisting of urea, yeast extract, and (NH_4_)_2_SO_4_ supported optimal bioflocculant production by *Virgibacillus* sp. Similarly, Gong et al. (2008) indicated that a mixed nitrogen source comprising urea and beef extract showed a substantial improvement on bioflocculant production by *Serratia ficaria* among others investigated.

Also, Kurane and Matsuyama (1994) reported on a bioflocculant produced from a mixed culture of *Acinetobacter*,* Agrobacterium*, and *Enterobacter* species in which the medium incorporated a combination of ammonium sulfate and yeast extract as the nitrogen source. Deng et al. (2005) documented that peptone and sodium nitrate were the best nitrogen sources among other sources tested for bioflocculant production by *A*. *parasiticus*. Li et al. (2013) noticed that peptone was more favorable for the production of bioflocculant by *Paenibacillus elgii* B69 among other nitrogen sources evaluated. Table 2 depicts the various optimum conditions for bioflocculant production, flocculating activity, chemical composition, and yields from different microorganisms.

**Table 2:** Optimum culture conditions, chemical compositions, flocculating activity, and yields of flocculating activity

Microorganism	Source	Carbon source	Nitrogen source	Chemical composition	Flocculating activity (%)	Yield	Citation
*Paenibacillus mucilaginosus*	Soil	Sucrose	Yeast extract	Polysaccharide	97	NA	Tang et al. (2014)
*Enterobacter aerogenes*	Soil	Fructose + glucose	Urea + Yeast extract + (NH_4_)_2_SO_4_	Glycoprotein	80	1.3	Lu et al. (2005)
*Oceanobacillus* sp. Pinky	Marine	Sodium carbonate	Tryptone	Glycoprotein	84.5	2.44	Cosa et al. (2013a)
*Chryseobacterium daeguense* W6	Backwashing sludge	Glucose	Tryptone	Glycoprotein	96.9	NA	Liu et al. (2010a)
*Paenibacillus polymyxa* BY‐28	Soil	Sucrose	Bean cake powder	Glycoprotein	99	NA	Gong et al. (2003)
*Corynebacterium glutamicum*	NA	Corn steep liquor	Urea + Corn steep liquor	NA	520 U/mL	NA	He et al. (2004)
*Virgibacillus* sp.	Marine	Glucose	Urea + Yeast extract + (NH_4_)_2_SO_4_	Polysaccharide	91.8	2.43	Cosa et al. (2013b)
*Aspergillus parasiticus*	NA	Starch	Peptone + Sodium nitrate	Glycoprotein	98.1	NA	Deng et al. (2005)
*Solibacillus silvestris*	Marine	Maltose	Yeast extract	Glycoprotein	90	1.7	Wan et al. (2013)
*Sorangium cellulosum*	NA	Soluble starch	NaNO_3_	Glycoprotein	96.6	17.5	Zhang et al. (2002)
*Klebsiella* sp.	Activated sludge	Glucose	Yeast extract + Urea	Polysaccharide	86.5	1.8	Yang et al. (2012)
*Klebsiella mobilis*	Soil	Dairy wastewater + Ethanol		Polysaccharide	95.4	2.58	Wang et al. (2007a,b)
*Penicillium purpurogenum*	NA	Glucose	Yeast extract	Polysaccharide	96	6.4	Liu and Chen (2010)
*Aeromonas* sp.	Activated sludge	Glucose	Peptone	Polysaccharide	92.4	2.25	Li et al. (2007)
*Serratia ficaria*	Soil	Lactose	Yeast extract	Polysaccharide	95.4	NA	Gong et al. (2008)
*Paenibacillus elgii* B69	Soil	Sucrose	Peptone + Yeast extract	Polysaccharide	87	25.63	Li et al. (2013)
*Rhodococcus erythropolis*	Activated sludge	Livestock waste water	NA	Glycoprotein	87.6	1.6	Peng et al. (2014)
*Bacillus licheniformis*	Contaminated LB medium	Sucrose	Yeast extract + Urea	Glycoprotein	700 U/mL	2.94	Xiong et al. (2010)
*Halomonas* sp.	Marine sediment	Glucose	Urea	Polysaccharide	95	NA	Mabinya et al. (2011)
*Klebsiella* sp. TG‐1	Wastewater	Sucrose	Beef extract	Glycoprotein	86.9	NA	Liu et al. (2013)
*Klebsiella pneumoniae*	Human saliva	Glucose	Peptone	Glycoprotein	96.5	4.7	Luo et al. (2014)
*Methylobacterium* sp.	Freshwater	Glucose	Urea + Yeast extract + (NH_4_)_2_SO_4_	Glycoprotein	95	8.203	Ntsaluba et al. (2011)
*Bacillus licheniformis* X14	Soil	Glucose	NH_4_Cl	Glycoprotein	99.2	NA	Li et al. (2009)
*Aeromonas* sp.	Activated sludge	Corn flour	Soyabean flour	Polysaccharide	49.34	NA	Li et al. (2008)
*Brachybacterium* sp.	Freshwater	Maltose	Urea	Glycoprotein	87.8	NA	Nwodo et al. (2012)
*Klebsiella* sp. ZZ‐3	Sludge	Glucose	NaNO_3_	Glycoprotein	92.6	0.126	Yin et al. (2014)
*Halobacillus* sp.	Marine sediments	Glucose	NH_4_Cl	Glycoprotein	93	0.34	Cosa et al. (2012)
*Bacillus clausii*	Brewery wastewater	Glucose	NA	Glycoprotein	88.67	NA	Adebayo‐Tayo and Adebami (2014)
*Vagococcus* sp.	Wastewater	Glucose	Urea + Yeast extract + (NH_4_)_2_SO_4_	Polysaccharide	86.5	2.3	Gao et al. (2006)
*Klebsiella pneumoniae*	Sputum	Glucose	Urea + Yeast extract + (NH_4_)_2_SO_4_	Glycoprotein	98	NA	Zhao et al. (2013)
*Citrobacter* sp. TKF04	Soil	Propionic acid and acetic acetic acid	Yeast extract	Glycoprotein	85	0.2	Fujita et al. (2000)
*Aureobasidium pullulans*	NA	Sucrose	NaNO_3_	Polysaccharide	NA	12.5	Ravella et al. (2010)
*Klebsiella* sp.	Activated sludge	Glucose	Yeast extract + Urea	Polysaccharide	86.5	3.52	Yang et al. (2012)
*Funalia trogii*	Laboratory	Maltose	Tryptone	Polysaccharide	NA	8.68	He et al. (2012)
*Enterobacter cloacae* WD7	Activated sludge	Glucose or sucrose	(NH_4_)_2_SO_4_	Polysaccharide	105	2.27	Prasertsan et al. (2006)
*Bacillus velezensis* 40B	Brackish water	Glucose	Yeast extract	Glycoprotein	99.9	3.54	Zaki et al. (2013)
*Bacillus alvei* NRC‐14	Soil	Chitosan	Yeast extract	Polysaccharide	98	10	Abdel‐Aziz et al. (2011)
*Halobacillus* sp. Mvuyo	Marine water	Glucose	Ammonium chloride	Glycoprotein	93	0.34	Cosa et al. (2012)
*Bacillus* sp. Maya	Marine	Glucose	Ammonium nitrate	Glycoprotein	95.6%	NA	Ugbenyen and Okoh (2013)
*Cobetia* sp. AOUIFE	Marine	Glucose	Urea + Yeast extract + (NH_4_)_2_SO_4_	Glycoprotein	92.78	NA	Ugbenyen et al. (2012)
*Bacillus* sp. Gilbert	Marine	Sucrose	Ammonium chloride	Polysaccharide	91	NA	Piyo et al. (2011)
*Halomonas* sp. Okoh	Marine	Glucose	Urea	Polysaccharide	95	NA	Mabinya et al. (2011)
*Arthrobacter* sp. Raats	Freshwater	Lactose	Urea	Glycoprotein	87.5	NA	Mabinya et al. (2012)
*Methylobacterium* sp.	Freshwater	Glucose	Peptone	Polysaccharide	72	NA	Ntsaluba et al. (2007)
*Micrococcus* sp. Leo	Marine	Glucose	Urea + Yeast extract + Urea	Glycoprotein	87.5	0.738	Okaiyeto et al. (2015a)
*Bacillus toyonensis* strain AEMREG6	Marine	Glucose	NH_4_NO_3_	Glycoprotein	89.5	3.2	Okaiyeto et al. (2015b)
*Bacillus* sp. AEMREG7	Marine	Glucose	Urea + Yeast extract + (NH_4_)_2_SO_4_	Glycoprotein		1.6	Okaiyeto et al. (2015c)
*Cellulomonas* sp. Okoh	Freshwater	Glucose	(NH_4_)_2_SO_4_	Glycoprotein	86.3	4.47	Nwodo and Okoh (2013)
*Streptomyces* and *Brachybacterium* species	Freshwater	Glucose	NH_4_NO_3_	Polysaccharide	63.7	3.02	Nwodo and Okoh (2013)
*Brachybacterium* sp.	Freshwater	Maltose	Urea	Glycoprotein	91.2	NA	Nwodo et al. (2012)
*Bacillus subtilis*	Soil	Cane molasses	Yeast extract	Polysaccharide	NA	4.92	Abdul‐Razack et al. (2014)

NA, not applicable.

## Effect of Metal ions on the flocculating activity of crude bioflocculants

Cations play a vital role in bioflocculation, in that they enhance the flocculating rate by neutralizing and stabilizing the residual negative charge/net surface charge of the functional groups on the bioflocculant, and thus, encouraging the formation of bridges between particles and the bioflocculant (Wu and Ye 2007). Cation plays a vital role in stimulating the adsorption of flocculants on suspended particles by lessening the distance between them and increasing the electrostatic attraction between the bioflocculant molecules and the suspended particles (Wang et al. 2011). Cosa et al. (2013a) found that calcium chloride and aluminum chloride were the most stimulating cations on the flocculation rate of the bioflocculant secreted by marine bacteria, *Oceanobacillus* sp. Pinky.

The cations were effective due to the protein nature of the bioflocculant which is rich in amino acids containing carboxyl groups that contribute to the negative charges of the particles; this produces a neutralization effect and bridge forming between the particles, thus allowing for improved bioflocculation. More carboxylate groups on the bioflocculant served as binding sites for the cations (Li et al. 2007). The addition of these cations to a suspension increased the floc size, resulting in enhanced sedimentation (Li et al. 2007). Banks et al. (2006) observed that the flocculating activity of a proteinous bioflocculant produced by *Rhodococcus erythropolis* and *Alcaligenes cupidus* was enhanced by the addition of Ca^2+^ and Al^3+^, respectively. Zheng et al. (2008) reported that the flocculating activity of the bioflocculant MBFF19 was increased in the presence of calcium ions, while Feng and Xu (2008) reported that the flocculating rate of the bioflocculant MBF3‐3 produced by *Bacillus* sp. was enhanced in the presence of the following metals: Mg^2+^, Al^3+^, Ca^2+^, K^+^, and Na^+^ ions, but inhibited in the presence of Fe^3+^ ions.

A similar finding in which Fe^3+^ completely inhibited the flocculating efficiency of the biopolymer secreted by *Bacillus* sp. F19 was also reported by Zheng et al. (2008). Contrary to the above observations, Wu and Ye (2007) reported that the flocculating rate of the bioflocculant p‐KG03 produced by *Gyrodinium impudicum* KG03 was improved in the presence of Fe^3+^ with a similar observation reported on the bioflocculant produced by *Enterobacter* sp. BY‐29 (Yokoi et al. 1997). Prasertsan et al. (2006) found that the bioflocculant produced by *Enterobacter cloacae* WD7 was stimulated in the presence of Cu^2+^. The novel biopolymers produced by *Citrobacter* sp. TKF04, *G. impudicum* KG30 and *Bacillus* sp. F19 required no cations for their flocculating activity (Fujita et al. 2000; Yim et al. 2007; Zheng et al. 2008). The flocculating efficiency of the bioflocculant produced by a haloalkaliphilic *Bacillus* species was drastically improved in the presence of divalent cations such as Ca^2+^, Cu^2+^, and Zn^2+^ (Kumar et al. 2004). Also, He et al. (2010) observed that the flocculation efficiency of the bioflocculant extracted from *Halomonas* sp. V3a’ was mediated by Ca^2+^ over a wide pH range of 3–11 resulting in flocculating activity of over 80% against kaolin suspension at a dosage of 4 mg/L.

The production of bioflocculants was influenced by the chemical nature of metal ions present in the culture medium (Li et al. 2009), with the bioflocculant produced by *Flavobacterium* sp. stimulated by Ca^2+^, Ba^2+^, and Mn^2+^ but subdued by the presence of Mg^2+^ (Gonzalez and Hu 1991). Li et al. (2009) reported that for strain X14, cations which included Na^+^, Ca^2+^, Fe^2+^, and Mg^2+^ had no effect on bioflocculant ZS‐7 production, while Cu^2+^ drastically inhibited cell growth. Cations can cause the neutralization of both the negative charges of the bioflocculant and suspended particles, thereby increasing the initial adsorption of a bioflocculant onto suspended particles (Wu and Ye 2007). The carboxylic functional groups of the bioflocculant provide the adsorption sites for cations (Prasertsan et al. 2006), thereby making the bioflocculant and kaolin clay particles form complexes. Lu et al. (2005) observed that the bioflocculant produced by *Enterobacter aerogenes* required Zn^2+^ for its flocculating activity, while Feng and Xu (2008) reported a synergic stimulation by Al^3+^, K^+^, Ca^2+^, Mg^2+^, and Na^+^ of the flocculating activity of the bioflocculant MBF3‐3 produced by *Bacillus* sp. Under optimized culture conditions, the flocculating efficiency of the biopolymer extracted from *S. ficaria* reached a maximum of 95.4% for kaolin suspension within a pH range of 5–7 with Ca^2+^ and Mg^2+^ serving as stimulants.

## Effect of Temperature and Agitation on the Production of Bioflocculants

Cultivation temperature has a great impact on bioflocculant production in microorganisms (Li et al. 2009). Enzymes responsible for bioflocculant production are activated at an optimum temperature (Zhang et al. 2007). From the literature search, the optimal temperature range for bioflocculant production varies between 25°C and 37°C (Salehizadeh and Shojaosadati 2001). The bioflocculant secreted by *Citrobacter* sp. TKF04 was cultivated at 30°C. Temperature had great influence on bioflocculant production, since optimum enzymatic reactions are usually attained at optimum temperature for microbial growth (Nakata and Kurane 1999). Shaker speed determines the concentration of dissolved oxygen that influences nutrient absorption and enzymatic reaction (Lopez et al. 2003). Li et al. (2009) reported that shaker speed of 140–160 rpm was optimal for the bioflocculant produced by *B. licheniformis* X14. Nonetheless, the disparity in shaking speed requirement for different microorganisms could be the result of the different oxygen requirements at different growth phases (Li et al. 2009).

## Effect of Inoculum Size on Production of Bioflocculants

Both Jang et al. (2001) and Gong et al. (2008) observed that the inoculum size among various physiological properties plays a substantial role in metabolic processes, in that it has a significant effect on cell growth and the production of secondary metabolites. A small inoculum size prolongs the stagnant growth phase; nevertheless, a large inoculum size causes the niche of the microorganism to overlap excessively, thereby suppressing bioflocculant production (Li et al. 2009). Cosa et al. (2013a) found that 2% inoculum size was optimal for bioflocculant production by *Oceanobacillus* sp. Pinky. Li et al. (2009) reported that 1% (v/v) inoculum size for X14 allowed the adaptation of strain X14 to the cultivation medium, decreasing the lag phase and promoting the production of bioflocculant ZS‐7. Studies by our group showed that the production of bioflocculant by *Micrococcus* sp. Leo was more propitious at 2% (v/v) inoculums size (Okaiyeto et al. 2014), while 3% (v/v) inoculums size was preferred for the production of bioflocculant by *Bacillus* sp. Gilbert (Ugbenyen et al. 2014).

## Effect of Initial pH of Production Medium on Bioflocculants Production

The initial pH of the fermentation medium is one of the factors that play a major role in the production of bioflocculant and also its flocculating efficiency (Zheng et al. 2008). It determines the electrification of the cells and oxidation–reduction potential which could influence the absorption of nutrients in the production medium and enzymatic reaction (Salehizadeh and Shojaosadati 2001). Mabinya et al. (2011) reported optimum bioflocculant production by *Halomonas* sp. OKOH at pH 7. Deng et al. (2003) reported that *Aspergillus parasiticus* preferred acidic conditions for synthesis, secretion as well as bioflocculant production in the fermentation medium, while *Klebsiellus* sp. TG‐1 preferred alkaline conditions of pH 8 for bioflocculant production (Liu et al. 2013). The bioflocculant secreted by *Halobacillus* sp. Mvuyo was more favorable at pH 7 (Cosa et al. 2012).

## Effect of pH on the Flocculating Activity of Produced Bioflocculants

When the pH of the medium is alkaline, the hydroxide ion (OH^−^) may obstruct the complex formed between the bioflocculant and the suspended particles mediated by metal ions and, consequently, lead to the suspension of suspended particles in the reaction mixture (Prasertsan et al. 2006). On the other hand, when the pH of the reaction mixture is adjusted to an acidic condition, the bioflocculant and the kaolin particles adsorb the H^+^ that weakens the complex formed between the bioflocculant and kaolin particles mediated by the metal ion, resulting in lower flocculating efficiency of the bioflocculant. The negative charge of the bioflocculant is believed to have resulted from the carbohydrate content, and the relevance of the proportion of protein to carbohydrates in determining the surface charge could be allied to the distinctive charge properties of proteins. Proteins consist of many amino acids which contain both carboxyl and amino groups and according to the observation of Liao et al. (2001), the amino groups from proteins possess positive charges which can neutralize some of the negative charges from both carboxyl and phosphate groups which in turn reduces the surface net charge (negative charge).

As with other organic acids, the carboxyl and amino groups ionize in aqueous solution. The molecule exists as a dipolar ion at a certain pH value, where both the acidic (acetic) and basic groups are ionized as zwitterions or hybrid ions (Liao et al. 2001). Prasertsan et al. (2006) found that the flocculating efficiency of the bioflocculant extracted from *E. cloacae* WD7 was optimal at pH 6.0, whereas Wang et al. (2011) noticed that the flocculating activity of the bioflocculant CBF‐F26 secreted by a mixed culture of *Rhizobium radiobacter* F2 and *Bacillus sphaeicus* F6 was maximal at neutral and weak alkaline circumstances. Deng et al. (2005) reported on the bioflocculant secreted by *A. parasiticus* at a pH range of 5–6. In addition, higher pH lowers biomass production as well as the flocculating activity of the produced bioflocculant. However, lower pH greatly improved fungal synthesis, secretion, as well as the accumulation of the bioflocculant in the fermentation medium (Deng et al. 2005). The bioflocculant produced by *Agrobacterium* sp. M‐503 maintained high flocculating activity at a pH range of 7–12 (Li et al. 2010). The flocculating efficiency of the biopolymer produced by *G. impudicum* KG03 was observed to be optimum within a pH range of 3–6 with optimum activity recorded at pH 4 (Yim et al. 2007).

## Cost‐Effective Substrates for Bioflocculants Production

In recent years, bioflocculants have gained huge scientific and biotechnological interest because of their degradability, the harmless nature of their breakdown products, and future application prospects (Nwodo et al. 2014). However, they have not been industrially applied because of their low flocculation ability in real wastewaters treatment, low yield, and high cost of production (Mabinya et al. 2012). The comparatively high cost of the commonly used substrates such as fructose, sucrose, galactose, maltose, and glucose has negative influence on production costs and this consequently restrict the market potential of these bioflocculants. One major measure to reduce the cost associated with the production of bioflocculants on an industrial scale was to employ low‐cost substrates (Fujita et al. 2000). Cheap substrates have been utilized for bioflocculant production (He et al. 2004; Xiong et al. 2010; Zhuang et al. 2012). Zhang et al. (2007) documented the utilization of brewery wastewater as a carbon source for bioflocculant production by a mixed culture of microorganisms.

Furthermore, efforts have also focused on the isolation of bioflocculant producing microbes competent in exploiting cost‐effective substrates and optimizing the media constituents and fermentation conditions in order increase bioflocculant yield (Sathiyanarayanan et al. 2013). Currently, response surface methodology (RSM), a statistical modeling is a promising tool that has been effectively applied to optimize bioflocculant production and this has provided consistent information that can be adduced for the optimization of bioflocculant production on a large scale (He et al. 2009; Li et al. 2013; Nwodo and Okoh 2014; Nwodo et al. 2014; Peng et al. 2014).

## Molasses as a Substrate

Molasses is a by‐product of the sugarcane industry that comprises approximately 50% (w/w) total sugars, vitamins, and nitrogenous compounds (Moosavi‐Nasab et al. 2010). The sugarcane molasses is a strong liquid with some distinctive features such as a high biochemical oxygen demand (BOD) concentration range (40,000–60,000 mg/L) and COD concentrations range (80,000–120,000 mg/L), and this liquid requires treatment before disposal in order to prevent environmental pollution. Zhuang et al. (2012 reported that the abundance of carbohydrates, amino acids, and proteins confers molasses with excellent properties for use as a possible substrate for bacterial growth culture and bioflocculant production.

Several bioflocculant‐producing microbes investigated hitherto utilize carbohydrate‐rich compounds as the sole source of carbon and energy (Li et al. 2009; Piyo et al. 2011; Ugbenyen et al. 2012). According to He et al. (2004), molasses is a cost‐effective nutrient that could be used as a high‐quality substrate by many microorganisms for the production of EPSs. Liu et al. (2010b) found that *Penicillium* sp. HHE‐P7 grows on molasses and that flocculating activity could reach 85% after 3 days of cultivation. *Pseudomonas alcaligenes* PS‐25 (Mao et al. 2010) and *Pseudomonas fluorescens* C‐2 (Mao et al. 2008) produced bioflocculants after 3 days of cultivation in molasses. He et al. (2004) reported that the bioflocculant REA‐11 production by *C. glutamicum* CCTCC M201005 was supported by the presence of glucose, fructose, and sucrose. Sucrose was preferred as the carbon source due to the lower cost and higher production rate of the bioflocculant (He et al. 2004). The ability to exploit sucrose makes it possible to utilize molasses as a carbon source for large‐scale production, thus making it possible to produce bioflocculants commercially. Sam et al. (2011) reported on the production of exopolysaccharides by halophilic bacteria which grew on a pretreated molasses as fermentation substrate.

## Brewery Wastewater as a Substrate

In breweries, the cleaning of tanks, bottles, machinery, and floors generates high quantities of contaminated water (Doubla et al. 2007). During brewing, large quantities of water are usually used and discharged into water bodies (Parawira et al. 2005; Simate et al. 2011). The discharge of untreated brewery wastewater may have a direct impact on water bodies (e.g., oceans, rivers, streams, or lakes) because the effluents are composed of organic compounds that need oxygen for degradation. For instance, when water of high organic matter content runs into a river, the microbes flora in the river tend to oxidize the organic matter, utilizing the available oxygen in the water quicker than the amount of oxygen dissolves back into the river from the air, thereby reducing the availability of oxygen for aquatic organisms (Simate et al. 2011). However, Chen et al. (2003) reported that, due to the availability of nutrient substances, brewery wastewater can perhaps be used as a good substrate for some microorganisms. Zhang et al. (2007) documented a novel bioflocculant produced by multiple microorganism consortia utilizing brewery wastewater as the sole carbon source. About 15 g of purified bioflocculant was recovered from 1 L of fermented culture. Under optimized culture conditions, the flocculating activity of the bioflocculant was about 96.8%. In addition, Rouka (1999) reported the production of Pollulan from brewery wastes by *Aureobasidium pullulans*.

## Diary Wastewater as a Substrate

Dairy industries produce various products such as ice cream, butter, milk, yoghurt, desserts of different kinds, and cheese which vary greatly in their characteristics that rely on the kind of system and methods of operation employed (Vidal et al. 2000). The dairy wastewaters usually have high BOD and COD concentrations, a representative of high organic content (Orhon et al. 1993). Perle et al. (1995) and Kasapgil et al. (1994) documented that dairy wastewaters are rich in nature, because of their high organic load which are supplied to these effluents from fats, proteins, and carbohydrates derived from the milk. Nonetheless, dairy wastewater is composed of a high concentration of organic matters which makes the effluents a serious threat to the local municipal sewage treatment systems (Perle et al. 1995).

Most of the wastewater volume obtained from the dairy industry comes from the cleaning of equipment in the production cycles, tank trucks, rinsing of milk silos, and equipment malfunctions or operational errors (Danalewich et al. 1998). According to Fang and Yu (2000), dairy wastewater is mainly composed of simple degradable carbohydrates, mainly lactose, with fewer biodegradable proteins and lipids. It can simply be described as a complex kind of substrate (Fang and Yu 2000). Demirel et al. (2006) revealed that lactose is the major carbohydrate in dairy wastewater and is a readily accessible substrate for the consumption anaerobic bacteria. The high carbohydrate contents in dairy wastewater were found to reduce the amount of proteolytic enzymes synthesized, resulting in low levels of protein degradation (Fang and Yu 2000). However, McInerney (1988) observed that carbohydrates may perhaps restrain the synthesis of exopeptidases, a cluster of enzymes assisting protein hydrolysis. Wang et al. (2007a,b) documented the production of a novel bioflocculant from a culture of *Klebsiella mobilis* using dairy wastewater supplemented with 2% (v/v) ethanol. By using the optimized culture conditions, 2.58 g of crude bioflocculant was extracted from 1 L fermentation broth and the flocculating activity was about 95.4%.

## Chemical Composition Analyses of Some Bioflocculants

Several studies have shown that most of the bioflocculants produced are either functional proteins (Zhang et al. 1999) or functional polysaccharides (He et al. 2004; Huang et al. 2005). Deng et al. (2003) documented that the bioflocculant MBFA9 secreted by *Bacillus mucilaginosus* was a polysaccharide composed mainly of amino sugar (2.7% w/w), uronic acid (19.1% w/w), and neutral sugar (47.4% w/w). The infrared spectrum analysis revealed the presence of carboxyl and hydroxyl as the major functional moieties. The flocculating efficiency of the biopolymer produced by *B. mucilaginosus* for kaolin suspension was about 99.6% at a dosage of 0.1 mg/L (Deng et al. 2003). Feng and Xu (2008) observed that the acidic bioflocculant produced by *Bacillus* sp. BF3‐3 is composed of polysaccharide (66.1% w/w) and protein (29.3% w/w).

The hydroxyl and carboxyl groups play a fundamental role in the flocculation of suspended particles because these functional groups provide adsorption sites where the suspended particles can be attached. Deng et al. (2005) documented the bioflocculant produced by *A.  parasiticus* utilizing corn starch as a carbon and peptone supplemented in the medium as a nitrogen source. This bioflocculant showed a high flocculating efficiency of 98.1% for kaolin suspension. The bioflocculant was able to flocculate dye from a suspension. The purified bioflocculant was composed mainly of sugar (76.3% w/w) and protein (21.6% w/w), and the average molecular weight was 3.2 × 10^5^ Da. The existence of amino and amide groups in the molecular chain might also influence the flocculation process (Deng et al. 2005).

The extracellular bioflocculants produced by a bacterium, a member of *Bacillus* genus, isolated from a Qatari ecosystem was able to flocculate kaolin suspension at a rate of over 85% at a dosage of 20 mg/L (Desouky et al. 2008). Also, Gao et al. (2006) found that the bioflocculant produced by *Vagococcus* sp. W31 was thermostable exhibiting a high flocculating efficiency in a wide pH range of 7–11 with a dosage requirement of 25 mg/L. The bioflocculant was mainly composed of polysaccharides with a molecular weight over 2 × 10^6^ Da and composed of neutral sugar (71.5% w/w) and uronic acid (15.4% w/w). The infrared spectra revealed the existence of hydroxyl, carboxyl, and methoxyl groups as major functional groups in the molecular chain of the bioflocculant. He et al. (2004) found that the bioflocculant produced by *C. glutamicum* was composed of polysaccharides and exhibited thermostability in an acidic pH range of 3.0–6.5.

The flocculating activity of the bioflocculant was relatively high compared to synthetic flocculants. These attributes indicate its potential utilization in the decolorization of molasses wastewater. The novel bioflocculant HBF‐3 produced by a deep‐sea bacterium mutant *Halomonas* sp. V3a’ was composed of a polysaccharide containing neutral sugar (20.6% w/w), uronic acid (7.6% w/w), amino sugar (1.6% w/w), and sulfate (5.3% w/w). The infrared spectra showed the existence of both hydroxyl and carboxyl groups in the molecular chain (He et al. 2010). Gao et al. (2006) found that the characteristics of the bioflocculant produced by any microbes are a predetermining factor that influences its flocculating efficiency. Also, in our research group, we recovered several bacterial isolates that produce bioflocculants which are polysaccharides (Mabinya et al. 2011; Piyo et al. 2011; Ntsaluba et al. 2013; Nwodo and Okoh 2014) and glycoproteins (Cosa et al. 2011; Cosa et al. 2012; Mabinya et al. 2012; Ugbenyen et al. 2012; Cosa et al. 2013a; Nwodo et al. 2013; Nwodo and Okoh 2013; Okaiyeto et al. 2014, 2015a).

## Important Properties of Bioflocculants

### Adsorption

Several reports have proven that the presence of carboxyl, amine, and hydroxyl groups in bioflocculants were the preferred functional groups required for flocculation process (Yim et al.^2007^; Wang et al. 2011; Eman 2012). According to He et al.'s (2010) findings, these functional groups are usually used for the adsorption process and which may serve as binding sites for metal ions and suspended particles. The presence of the carboxyl groups on the molecular chain of the biopolymer allows the chain to spread out as a result of electrostatic repulsion and the stretched molecular chains provide more effectual sites for particle attachment (Pathak et al. 2014). The amino and carboxyl functional groups of bioflocculant can form a complex with heavy metals by neutralizing and stabilizing the residual charge as the binding distance is shortened (Yue et al. 2006).

This adsorption property of bioflocculantals shows the prospective roles of bioflocculant in heavy metal sorption to bacterial cells and transporting in environments (Hu et al. 2007). The adsorption capability of the bioflocculant depends on the numbers of the available carboxyl and hydroxyl groups (Sheng et al. 2010). The presence of numerous functional groups, for example, in case of glycoproteins which contain both functional group of carbohydrate and protein accounts for its high binding capacity (Guibaud et al. 2003). Due to the extensive capacity of bioflocculants for metals, they are recommended as surface‐active agents for the removal of heavy metals (Pathak et al. 2014). The physical and chemical properties of the metals, the availability of appropriate binding sites present to metal, as well as the tertiary structure of bioflocculant may all contribute to metal‐binding interactions (Kachlany et al. 2001).

Bioflocculants that are predominantly composed of protein have lower adsorption capability compared to carbohydrate bioflocculants or glycoprotein bioflocculants with several adsorption sites. The bioflocculants are always negatively charged, and this feature is advantageous in the binding of positively charged organic pollutants since they offer cation exchange potential through electrostatic interaction (Esparza‐Soto and Westerhoff 2003). Several bioflocculants have been reported in the previous studies from pure strains with strong adsorption capability for metal ions (Gao et al. 2009; Lin and Harichund 2011; Eman 2012; Rawat and Rai 2012; Batta et al. 2013; Li et al. 2013). The metal biosorption by biopolymer involves physical–chemical interactions between the metal and the functional groups of the bioflocculant. This biosorption involves several mechanisms, including physical adsorption, complexation, ion exchange, and precipitation (Wingender et al. 1999). The effectiveness of biosorption by bioflocculant depends on the pH, temperature, effective contact area between bioflocculant and adsorbate, time of contact, ionic strength, and concentration of the adsorbate, adsorbate structure, and the type of microorganism (Solis et al. 2012).

### Biodegradability

In batch culture fermentation, the bacterial cell increases as a result of abundant of nutrient (Nwodo and Okoh 2013). The secondary metabolite (bioflocculant) released into the medium by the bacteria are mainly composed of carbohydrates and proteins. When the nutrient is in shortage, the bacteria degrade the bioflocculant produced inside the environment due to their biodegradability property as sources of carbon and energy for cell growth (More et al. 2014). Equally, when bioflocculants are used in wastewater treatment reactors, the enzymes responsible for their degradation are usually in abundant (Sheng et al. 2010). In addition, the bacteria in activated sludge can utilize the biopolymers that are excreted by other bacteria for metabolic activity (Zhang and Bishop 2003). Bioflocculant degradation can also result in the deflocculation of sludge flocs. Generally, this biodegradability property of bioflocculant can be linked to the reason why they cannot instigate environmental pollution unlike PAAs which are not biodegradable and hence constitute environmental nuisance (Shih et al. 2001).

### Hydrophobicity/hydrophilicity

Hydrophobicity is very important property of the bioflocculant. Hydrophobicity results from the behavior of bioflocculant particles or molecules, which are incapable of interacting electrostatically or establishing hydrogen bonds with water, induce hydrophobic properties with bioflocculant (More et al. 2010). The bioflocculant comprises numerous charged functional groups such as carboxyl, phosphoric, sulfhydryl, phenolic, and hydroxyl groups and nonpolar groups such as aromatics, aliphatics in proteins, and hydrophobic regions in carbohydrates (Flemming and Leis 2003). The formation of hydrophobic areas in bioflocculant would be beneficial for organic pollutant adsorption (Spath et al. 1998). The presence of hydrophilic and hydrophobic groups in bioflocculant molecules shows that bioflocculants are amphoteric in nature. It also demonstrates the importance of the bioflocculant as the sorption sites for organic pollutants (Flemming and Leis 2003). The hydrophilicity/hydrophobicity of bioflocculant is likely to significantly influence the hydrophobicity of microbial aggregates and their formation in bioreactors (Liu and Fang 2002).

## Factors Influencing the Flocculating Activity of Bioflocculant

### Effect of dosage

Dosage requirement is still one of the most critical factors to be considered when determining the optimum conditions for the performance of bioflocculant in the process of coagulation/flocculation, since an insufficient dosage or overdosage may lead to reduced performance in flocculation (Hassan et al. 2009). Hence, it has becomes essential to establish the optimum bioflocculant dose, as this could help minimize costs and attain better performance in the treatment processes (Cosa and Okoh 2014). However, it has been stated in the literature that an insufficient bioflocculant dosage might not be appropriate for the neutralization of the negative charges on kaolin particles (Li et al. 2007). In addition, the settling of flocculated particles can be negatively affected due to the high viscosity from the excessive level of bioflocculant molecules in the solution (Yim et al. 2007; Wang et al. 2011).

The optimum bioflocculant dose for the purified bioflocculant was 0.8 mg/mL with a resultant flocculating activity of 90%. Wang et al. (2011) similarly reported that the bioflocculant CBF‐F26 produced from a mixed culture of *R. radiobacter* F2 and *B. sphaeicus* F6 at a bioflocculant dosage of 12 mg/L showed a maximum flocculating activity of 96%. Flocculating activity of MBF3‐3 produced by *Bacillus* sp. was highly improved as the dosage increased from 0.25 to 4.0 mg/L. When MBF3‐3 dosage was 4.0 mg/L, flocculating activity reached a maximum value 96.9. However, the flocculating activity decreased with higher MBF3‐3 dosages (Feng and Xu 2008). The flocculating activity of biopolymer flocculant secreted by *Klockera* sp. was over 94% in the dosage range of 0.00425–0.013 mg/mL and attained its highest flocculating rate of 98.13% at 13 mg/mL (Abu‐Elreesh et al. 2011).

In the case of *Bacillus mojavensis*, the cost‐effective bioflocculant dosage was 0.003 mg/mL, which resulted in flocculating activity of 89.7% at pH 7 (Elkady et al. 2011). Flocculating activity of MBF‐6 produced by *K. pneumoniae* YZ‐6 was over 80.0% in a range of MBF‐6 dosages of 30.0–90.0 mg/L, with the maximum flocculating activity being observed in an optimal dosage of 50.0 mg/L (Luo et al. 2014). Okaiyeto et al. (2013) reported that highest flocculation rate was achieved at lower bioflocculant concentrations of 0.2 mg/mL for the purified bioflocculant produced by a mixed culture of *Halomonas* sp. Okoh and *Micrococcus* sp. Leo. On the contrary, Zhao et al. (2013) found that flocculating rate of the bioflocculant γ‐PGA produced by *B. licheniformis* decreases at concentrations below or above 1.5 mg/L, whereas, in the case of the bioflocculant produced by *Corynebacteria daeguense*, the optimal concentration that was favorable for the flocculating activity of the bioflocculant was 1.2 mg/L (Liu et al. 2010a).

When the bioflocculant dosage is insufficient, the bridging phenomena cannot be effectively formed. On the other hand, excessive dosage of bioflocculant may cause competition and repulsion of negatively charged particles, consequently blocking the sites available on the particle surfaces for the formation of interparticle bridges and thereby leading to restabilization of the kaolin particles in suspension and hence, a decrease in the flocculating efficiency of the bioflocculant (Gong et al. 2008; Sun et al. 2012; Guo et al. 2014).

According to Liang et al. (2010), the decrease in flocculation activity that occurred may be attributed to “flocculation deterioration” phenomenon whereby some colloidal particles were encased by the concentrated flocculant and a “colloid protection function” occurred, leading to reduced flocculating activity. The binding sites of the dispersive kaolin particles were blocked up by some bioflocculant molecules at high bioflocculant dosage instead of the formation of stronger bridging among the bioflocculant molecules and disperse particles in a proper flocculant dosage (He et al. 2010). This hypothesis is premised on the assumption that a three‐dimensional matrix model is formed between disperse matters and extended polymer chains in terms of the bridging phenomena with the help of intermolecular force, such as van der Waals’ force and hydrogen bond. It was difficult to coagulate and bridge when the bioflocculant was insufficient (Zhang et al. 2010). On the contrary, super abundant bioflocculant would mask the disperse particles, and block the formation of bigger flocs (Lu et al. 2005).

### Effect of cations

In accordance to Salehizadeh and Shojaosadati (2001) investigation, bioflocculants cause aggregation of cells and particles by bridging and charge neutralization. The flocculation of negatively charged kaolin particles by anionic bioflocculant may be made possible by cationic bridge formation between particles and bioflocculant chains (Wu and Ye 2007). Flocculation occurs as a result of a decrease in the negative charge on the particles’ surface in the presence of cations, and this consequently reversed the net surface charge on the kaolin particles from negative to positive. The cations could stimulate the flocculation by neutralizing and destabilizing residual negative charges of carboxyl groups of uronic acid in an acidic polysaccharide, forming bridges which bind kaolin particles to each other (Liu et al. 2010a).

The property and structural components of bioflocculants are highly dependent on their microbial origin as the metal ions exhibit varying effects on different bioflocculants, as their enhancing effects depend on both the valence and concentration of the cations (Wu and Ye 2007). This means that concentration and valence of metal ions play significant roles in destabilizing of colloid systems. Cations can neutralize the negatively charged kaolin suspension and cover the adsorption sites of bioflocculants through bridging mechanisms (Yim et al. 2007; He et al. 2010).

Bioflocculant is essentially a kind of polymer which is usually negatively charged. However, this characteristic limits the application of bioflocculant in water treatment because most water pollutants are negatively charged as well (Huang et al. 2014). Thus, in order to extend the application of bioflocculant, researchers have used it in combination with conventional coagulants for water treatment. It has been well documented that to achieve high flocculating activity, metal ions are usually required to aid the flocculation process (Salehizadeh and Shojaosadati 2002; Gong et al. 2008; Elkady et al. 2011). Specifically, the cation is used as coagulant aid in achieving high flocculation activity by neutralizing the negatively charged functional groups on the bioflocculant and suspended particles thereby increasing the adsorption of bioflocculant to the suspended particles (He et al. 2010; Mabinya et al. 2011).

Several studies have been documented in the previous studies on the synergistic effects of cations on different bioflocculants. For example, in the case of the bioflocculant produced by *Serratia ficaria*, the flocculating activity was enhanced by the addition of Ca^2+^ and Mg^2+^, whereas Al^3+^ and Fe^3+^ showed a negative effect (Gong et al. 2008). The bioflocculant produced by *Halomonas* sp. and *Micrococcus* sp. was cation dependent with improved flocculating activity in the presence of Al^3+^, Ca^2+^, and Mn^2+^ and inhibited by Ba^2+^, Mg^2+^, Fe^3+^, Na^+^, Li^+^, and K^+^ (Okaiyeto et al. 2013). In the case of a bioflocculant produced by *Virgibacillus* sp. Rob, monovalent cations (Na^+^, Li^+^, K^+^) and the trivalent cation Fe^3+^, showed little effect on flocculation activity, whereas divalent cations (Ca^2+^, Mn^2+^, Mg^2+^) and Al^3+^ greatly improved flocculating efficiency of the bioflocculant (Cosa et al. 2013c). As the flocculation process proceeds, the charge bridging between the bioflocculants and the kaolin particles leads to an increase in floc density, floc size, and the floc resistance to shear. However, with the observation of Cosa et al. (2013c), the monovalent cations showed little synergistic effect due to reduction in the strength of the bonds that consequently cause a loose structure of flocs, and thus resulting in a decrease in floc density, size, and floc resistance to shear. This explains why the trivalent and bivalent cations have stronger synergistic effect for flocculation (Wu and Ye 2007).

Similarly, Salehizadeh and Shojaosadati (2002) and Elkady et al. (2011) reported an analogous findings where monovalent cations showed weak stimulation of flocculation by their respective bioflocculants. The bioflocculant produced by *Brachybacterium* sp. required Ca^2+^, Mg^2+^, and Mn^2+^ for effective flocculation (Nwodo et al. 2012), whereas the flocculating activity of the bioflocculant produced by *Bacillus velezensis* was stimulated in the presence of Ca^2+^, Zn^2+^, and Na^+^ and inhibited in the presence of Al^3+^, Fe^3+^, and Mg^2+^ (Zaki et al. 2012). The surfaces of kaolin particles were strongly negatively charged, divalent cation Ca^2+^ could compress the double layer of kaolin particles, weaken the static repulsive force, and promote HBF‐3 to form floc with kaolin particles (He et al. 2010). Charge neutralization happened when suspended particles were oppositely charged against the bioflocculant. In this case, surface charge density of the suspended particles was reduced by the adsorption of the bioflocculant and the particles can approach sufficiently close to each other so that the attractive forces become more effective (Li et al. 2009). As most bioflocculants and suspended particles are negatively charged, charge neutralization seldom occurs in the flocculating process (He et al. 2010).

On the other hand, the addition of metal ions had no effects on flocculating activity of MBF‐7, indicating that MBF‐7 was cation‐independent (Zhong et al. 2014). Similarly, the flocculating activity of bioflocculants, p‐KG03 and MBF‐6, produced by *G. impudicum* KG03 and *K. pneumoniae* YZ‐6, respectively, were not enhanced by the addition of any cation (Yim et al. 2007; Luo et al. 2014). Also, the addition of metal ions had no positive effects on the flocculating activity of MBF‐6 produced by *K. pneumoniae* YZ‐6, indicating that MBF‐6 was cation independent, and it could avoid second pollution and reduce cost. The bioflocculants produced by *A. flavus* and *K. pneumoniae* were cation independent, which showed an outstanding performance in kaolin clay suspension without the addition of metal ions (Aljuboori et al. 2013; Zhao et al. 2013).

### Effect of pH

pH is one of the most important external factors affecting flocculating activity of bioflocculants (Salehizadeh and Yan 2014). The pH of reaction mixtures is a key factor influencing the flocculation process (Zaki et al. 2013). Literature suggests that the alteration of pH may ultimately alter the bioflocculant charge status and surface characteristics of suspended particles consequently changing the flocculating ability (Zhang et al. 1999). This variation in the pH requirement of the reaction mixture may be due to the bioflocculants showing different electric states at different pH values and hence affecting the flocculation capability of the bioflocculants for the kaolin particles (Pan et al. 2009).

The flocculating activity of bioflocculant from *P. elgii* was over 80% in a wide range of pH from 3 to 11, and the bioflocculant was pH stable, indicating its wide range of field applications (Li et al. 2013). In addition, one of the ways that pH influences flocculating activity is by affecting the stability of suspended particles and the formation of floccules (Ugbenyen et al. 2014). However, it has been demonstrated that at very high pH, the OH^−^ ions may impede the formation of the complex between the bioflocculant and kaolin particles in the mixture. The purified MBF‐7 had an optimum pH of 5 with small noticeable differences in flocculating activity in the pH range of 3–6, whereas at higher pH of 7–12, flocculating activity decreased gradually (Zhong et al. 2014). In basic solutions (pH 9–12), the flocculating activity decreased gradually (from 74% down to 21%) due to alkaline degradation of the polysaccharide which could cause several changes such as molecular rearrangement of its residue or fragmentation of the polysaccharide chain (Zhong et al. 2014). It might also be that the hydroxide ion (OH^−^) absorbed at basic condition interferes with the complex formation of the polysaccharide and kaolin particles, consequently the kaolin particles were suspended in the mixture.

The bioflocculant of *G. impudicum* KG03 was active in acidic conditions ranging from pH 3 to 6, with the maximum activity observed at pH 4 (Zhang et al. 2002). On the other hand, He et al. (2010) reported that the flocculating activity of HBF‐3 held more than 80% in the pH range and the peak flocculating activity 97.0% occurred at pH 7.0. At low pH, both HBF‐3 and kaolin particles were likely to absorb hydrogen ions (H^+^), which weakened the forming of complexes between HBF‐3 molecules and kaolin particles mediated by Ca^2+^. Similarly, hydroxide ions (OH^−^) interfered with the combination of the bioflocculant molecules and kaolin particles at high pH, resulting in lower flocculating activity.

The bioflocculant produced by *Ruditapes philippinarum*, showed a high flocculating activity in a wide pH range from 1 to 13, with the optimum pH in the range of 7–9 (Gao et al. 2009). The flocculating activity of bioflocculant produced by *C*. *daeguense* was recorded at more than 90% within the pH range of 4–8 with the highest flocculating activity of 96.8% at pH 5.6, which subsequently decreased in flocculating activity out of this pH range (Liu et al. 2010a). Bioflocculant produced by *Bacillus* sp. UPMB13 has a relatively wide pH tolerance ranging from slightly acidic to slightly alkaline condition. The result shows that the bioflocculant can perform at pH ranges from 4.0 to 8.0 (Zulkeflee et al. 2012). The negatively charged density of the bioflocculant rose with increasing pH, which further increased the electrostatic repulsion of the negatively charged kaolin particles, and thus, poor flocculating activity was observed (Guo et al. 2015).

### Effect of temperature

The thermal stability of bioflocculant is an important property for its commercial exploitation (Marinho‐Soriano and Bourret 2005). Several studies on thermal stability of bioflocculants produced by different organisms have been documented in the literature (Gong et al. 2008; Gao et al. 2009; Wang et al. 2013; Ugbenyen and Okoh 2014). The exhibition of thermal stability by these bioflocculants may be characteristic of their polysaccharide backbone (Lu et al. 2005). The bioflocculant produced by *O*. *ciceri* maintained flocculating activity of kaolin suspension at over 90% in the temperature range of 30–90°C, but sharply decreased at temperatures above 90°C (Wang et al. 2013). The bioflocculant produced by *A*. *flavus* was thermostable over acidic and neutral pH values, and over 90% of flocculating activity was maintained within the temperature range of 10–100°C (Aljuboori et al. 2013). The MBF‐6 produced by *K. pneumoniae* YZ‐6 showed strong flocculating activity over a broad range of temperature (0–70°C), and it maintained excellent flocculating activity at lower temperatures, exhibiting its great application potential in treating low temperature water (Luo et al. 2014).

Furthermore, the bioflocculant produced by *C. glutamicum*, which retained high flocculating activity of 96.9% at 80°C, but the stability decreased slightly on increasing the temperature to 100°C (Liu et al. 2013). The bioflocculant produced by *K. pneumoniae* flocculated well in the range of 4–50°C with flocculating activity of above 88%, and the highest flocculating activity of 97.5% was achieved at 30°C. Flocculating activity decreased slightly, when the temperature exceeded 30°C might be due to the denaturation of proteins in the bioflocculant and an increase in hot movement of kaolin particles (Liu et al. 2010a). If temperature is too high, although reaction speeds up, the formed floces are too small and have stronger hydrating trend, and as a result, it was difficult to be separated by precipitation. If temperature is too low, reaction slows down, the increase of shear intensity of water to flocculants makes flocs too small to be separated by precipitation (Pan et al. 2009). On the other hand, Salehizadeh et al. (2000) reported a less stable bioflocculant that lost about 50% of the flocculating activity after heating for 15 min at 100°C.

### Effect of chemical compositions and molecular weight of bioflocculant

The chemical composition of bioflocculant is an important factor which determines its flocculating activity. Most of the reported bioflocculants in the previous studies predominantly composed of polysaccharides, proteins, fatty acids, and nucleic acids (Salehizadeh and Shojaosadati 2003). The functional groups of the bioflocculants provide adsorption sites for different suspended particles. The binding capability of the bioflocculants depends on the number of functional groups in their molecular chains. The surface charge and hydrophobicity of the bioflocculant are important in sludge settling. The important factor determining the charge of the bioflocculant is the proportion of carbohydrates to protein in the bioflocculant.

For example, Wan et al. (2013) reported that the chemical analysis of the purified bioflocculant produced by *S. silvestris* W01 indicated that it is a proteoglycan composed of 75.1% carbohydrate and 24.9% protein (w/w), whereas in the case of bioflocculant produced by *Klebsiella* sp. ZZ‐3, the composition of ZZ‐3 was found to be 84.6% polysaccharides and 6.1% protein. Deng et al. (2005) reported that the bioflocculant produced by *A. parasiticus* could be used to remove dye and composed of carbohydrate (76.3%) and protein (21.6%). Zheng et al. (2008) reported a bioflocculant produced by *Baccillus* sp. F19 which was composed of neutral sugar (3.6% w/w), uronic acid (37.0% w/w), amino sugars (0.5% w/w), and protein (16.4% w/w). He et al. (2010) investigated the novel bioflocculant HBF‐3 produced by a deep‐sea bacterium mutant *Halomonas* sp. V3a’ and found it to be composed of mainly a polysaccharide (29.8%) including neutral sugar residues (20.6%), uronic acid (7.6%), amino sugar (1.6%), and a sulfate group (5.3%).

Bioflocculants having negative surface net charge show higher hydrophobic and these hydrophobic components form bonds with positively charged inorganic particles such as metal ions and dye molecules (More et al. 2010). Polysaccharides play the major role in flocculation. For example, Bruus et al. (1992) suggested that the divalent cations interact with negatively charged groups of alginate like polysaccharides within bioflocs. Multivalent cations (divalent, trivalent) may bridge among negatively charged carboxyl groups (of uronic acids) (Subramanian et al. 2009). In addition, it is important to note that the efficiency of the bridging mechanism in flocculation can be related to the size of the bioflocculant. Flocculation process involving bioflocculants of high molecular weight involves more adsorption points, stronger bridging, and higher flocculating activity than flocculation with involving a low‐molecular‐weight bioflocculant (Zhang et al. 2010).

Several bioflocculants of high molecular weight from pure strains have been documented in the previous studies (Yim et al. 2007; Xiong et al. 2010; Tang et al. 2014). For example, the molecular weight of the bioflocculant produced by *Paenibacillus elgii* B69 was about 3.5 × 10^6^ Da based on the calibration curve with standard dextran. Also, the molecular weight of bioflocculant from *B. licheniformis* X14 was only 6.89 × 10^4^ Da (Li et al. 2009), whereas the one extracted from *Klebsiella* sp. ZZ‐3 had a molecular weight range of 603–1820 kDa (Yin et al. 2014). The average molecular weight of a novel bioflocculant produced by *B. licheniformis* was approximately 1.76 × 10^6^ Da (Xiong et al. 2010), whereas Li et al. (2010) documented that the molecular weight of a thermal and alkaline stable biopolymer produced by *Agrobacterium* sp. M‐503 was 8.1 × 10^4^ Da which was a significant factor in aiding the bridging mechanism in flocculation in kaolin suspension.

## Flocculation Mechanisms of Bioflocculant

Although the mechanism of flocculation by chemically synthesized flocculants is well described in the literature, the mechanism of flocculation by the biopolymers secreted during growth of microorganisms is still yet to be fully studied and understood (Salehizadeh and Shojaosadati 2001). Bridging and charge neutralization have been used to explain the mechanism of flocculation in biological systems based on their experimental observations (Lian et al. 2008; Li et al. 2009; Wang et al. 2011).

## Flocculation Mechanism by Bridging

Bridging in biological systems occurs when a bioflocculant forms threads or fibers in solution, they usually stretch out like a branch of a tree and extends from the particles’ surface into the solution for a distance greater than the distance over which the interparticle repulsion acts and thereby making flocculation to be effective (Salehizadeh et al. 2000). In this case the bioflocculant molecules attract suspended particles, making them to come together as aggregates (Li et al. 2008). The effectiveness of the bridging mechanism depends on the molecular weight and the net charge of the bioflocculant, the suspended particle, the ionic strength of suspension, and the nature of mixing (Wang et al. 2011; Yuan et al.^2011^). Bioflocculants with higher molecular weight mean longer molecules containing high functional groups and this implies effective bridging because they usually have more attractive adsorption sites (Zhang et al. 2010). When a bioflocculant molecule comes into contact with a suspended particle, some of the reactive groups on the bioflocculant adsorb at the particle surface, leaving other portions of the polymer molecule extending into the solution (Yim et al. 2007). If excess polymer is added or adsorbed, the particles are restabilized by surface saturation and are statically stabilized (Li et al. 2008). For example, bridging mechanism was found to play a key role in flocculating efficiency of the bioflocculants EPSSM9913 produced by *Pseudoalteromonas* sp. SM9913 and ZS‐7 from *B. licheniformis* X14 (Li et al. 2008; 2009), *Bacillus megaterium* TF10 (Yuan et al. 2011), and *Proteus mirabilis* TJ‐1(Xia et al. 2008).

## Flocculation Mechanism by Charge Neutralization

When negatively charged particles are in aqueous solution, they move continuously exhibiting Brownian movement. Since charged particles exhibit electrostatic repulsion forces which are greater than the van der Waals forces of attraction between them, they inhibit their settling and floc formation (Lachhwani 2005). For particles in aqueous solution to settle, an opposite charged compound usually a positively charged flocculant is required in order to neutralize and stabilize the negative charge of suspended particles (Salehizadeh and Shojaosadati 2001). When flocculants are added, flocs are usually formed and this hastens the gravitational settling of particles in solution. Initially the flocculation process involves the formation of small flocs which later aggregate to form a larger floc thus speeding up the sedimentation rate (Lachhwani 2005). The particle surface charge is reduced when it is adsorbed onto the bioflocculant leading to increased attractive forces compared to repulsive forces (Levy et al. 1992). Many researchers have reported this flocculation mechanism in many bioflocculants produced by different microorganisms. Levy et al. (1992) stated that when the bioflocculant is oppositely charged compared to the suspended particles, the particle surface charge density is reduced by its adsorption onto the bioflocculant causing the particles to approach sufficiently close to each as attraction forces become more effective than repulsive forces. Adsorption of the particles by the polymers occurs as a result of uneven distribution of charges (Salehizadeh and Shojaosadati 2001).

## Application of Extracellular Polymeric Substances

Recently, the exploration of potential EPS utilization has increased tremendously because of its numerous unique properties that suggest its potential applications in various industrial processes (Vu et al. 2009; Elkady et al. 2011). Due to their unique biophysicochemical properties, EPS are used in various industrial processes, for example, in the production of cosmetics, textiles, adhesives, detergents, pharmaceuticals, food additives, as well as brewing (Liu et al. 2010b; Mishra and Jha 2013). Furthermore, EPS could serve as bioflocculants, antioxidant, heavy metal removal, a natural immunomodulator, drug delivery agents in wastewater treatment, oil recovery, dredging, and in diverse downstream processes (Wang et al. 2008). Some of these biopolymers have also been reported to have antiviral, anti‐inflammatory, antitumor properties, serve as inducers for interferons, and colony‐stimulating systems (Lin and Zhang 2004). Among biopolymers identified, polysaccharides draw the attention of researchers in the field of flocculation predominantly in water treatment (Crini 2005; Raza et al. 2011).

The production of polysaccharide‐rich bioflocculants is not species specific, and it is possible that each strain of the same species secretes diverse kinds of polysaccharides in the cultivation medium with different biological functions (Sathiyanarayanan et al. 2013). Polysaccharides possess hydroxyl groups, with a hemiacetal reducing end, as well as other functionalities that play essential roles in reduction reactions (Mata et al. 2009). Kunmani et al. (2011) found that EPSs can be used in the food industry as viscosifying, stabilizing, and emulsifying agents. These compounds have been of interest as antitumor, antiviral, and anti‐inflammatory agents and as inducers of interferon, platelet aggregation inhibitors, and in colony‐stimulating factor synthesis utilized in various medical and pharmaceutical industries. Bioflocculants have been extensively used in wastewater treatment, for example, in the treatment of dye solutions (Zhang et al. 2002; Deng et al. 2005), inorganic solid suspensions (bentonite, solid clay, alumimum oxide, Ca(OH)_2_, and activated carbon) (Levy et al. 1992; Shih et al. 2001; Yim et al. 2007).

## Purification of Wastewater, COD, and Suspended Solids Removal

The bioflocculant produced by *P*. *elgii* B69 showed high flocculating ability in purifying different wastewaters which included COD removal (68%), turbidity reduction (83%), and color removal (88%) (Li et al. 2013). Gong et al. (2008) also reported on the bioflocculant produced by *S. ficaria* with the high flocculating activity of kaolin suspensions as well as showing good flocculating efficiency in different wastewaters. River water is surface water characterized with low COD and turbidity. Compared with chemical flocculants in the clarification of river water, bioflocculant SF‐1 produced by *S. ficaria* had a better flocculating activity, with the removal efficiency of 87.1% for COD, 84.2% for turbidity and 90.4% for color (Gong et al. 2008). Furthermore, bioflocculant SF‐1 produced by *S. ficari*a was also used to flocculate different wastewaters. The COD removal for brewery wastewater was 80.7%, turbidity removal was 91.8%, whereas for meat processing wastewater, the COD removal was 76.3%, turbidity removal was 93.7% and for soy sauce brewery wastewater color removal of 64.1% (Gong et al. 2008). The bioflocculant MBFA9 produced by *B. mucilaginosus* had a strong flocculating activity for suspended solids with a removal rate of 85.5% and the COD removal rate of 68.5% (Deng et al. 2003). Luo et al. (2014) also reported on bioflocculant MBF‐6 produced by *K. pneumoniae* YZ‐6 isolated from human saliva with the ability to flocculate several wastewaters from the textile, dairy, brewery, and the sugar industries. The maximum flocculating efficiency observed with wastewater from the sugar industry showed the highest reduction of COD (77.8%) and BOD (80.7%). In addition, the bioflocculant reduced the suspended solids by up to 78.6% (Luo et al. 2014). The bioflocculant produced by *B. mucilaginosus* had a COD removal rate of 74.6% and 42.3% for BOD for domestic wastewater with the removal rates for brewery wastewater recorded at 70.5% and 77.4% for COD and BOD, respectively. Furthermore, its removal rates of COD and BOD for pharmaceutical wastewater was 66.2% and 41.7%, respectively (Lian et al. 2008). Gong et al. (2008) found that the bioflocculant produced by *S. ficaria* had a good COD removal capability and decolorization of pulp effluent than traditional chemical flocculants.

In addition, a number of studies have demonstrated the efficiencies of bioflocculants in the removal of suspended solids, latex particles, microorganisms, COD, humic acids, heavy metals from waste streams, as well as separation of oil from oil–water emulsions and fine coal processes (Ma et al. 2008; Zemmouri et al. 2011). Most bioflocculants produced in the literature have demonstrated good flocculating activity for kaolin suspension. Nevertheless, they show different flocculating ability for other suspended particles in aqueous solution. Deng et al. (2003) found that a polysaccharide‐rich bioflocculant exhibited an excellent flocculating potential in the recovery of the organic solids from starch wastewater, while Kurane et al. (1991) reported on the bioflocculant produced by *Aspergillus latus* that was able to flocculate oil emulsion.

## Heavy Metal Removal

Quite a number of industrial processes resulted in the discharge of heavy metals into aquatic ecosystems. This has necessitated appropriate attention because of the negative impacts of these heavy metals on the environment (Salehizadeh and Shojaosadati 2003). The problem associated with heavy metals in wastewater entering natural waters has been well documented (Florence and Morrison 1992). Inorganic effluents from the industrial processes comprise harmful metals such as Cd, Zn, Cr, Ni, and Cu (Kurniawan 2002), which tend to build up in the food chain, and their pollution of fresh water is a cause for great concern (Cobbing 2008). The metals tend to sediment to the bottom of the freshwater where they concentrate and are capable of accumulating in the tissues of aquatic biota. However, their high solubility in aquatic environments means that they can be assimilated by living organisms and once they enter the food chain, they are bound to also manifest in humans. If the metals accumulate in the body above their limit, they can pose severe health problems. Hence, it is essential to treat metal‐contaminated wastewaters before their release to the environment (Kurniawan 2002). The escalating crisis of heavy metal pollution of soil, water, and some other sediment has made seeking for alternatives to eliminate these contaminants a priority. Recently, studies on heavy metal removal from wastewater and coal have spotlighted the development of materials that have increased affinity, capacity, and selectivity for target metals (Pazirandeh et al. 1998). The removal of toxic heavy metals from industrial wastewaters is essential from the standpoint of environmental pollution control (Guangyu and Thiruvenkatachari 2003). The use of bacteria capable of producing compounds such as extracellular polysaccharide (EPS) and cell wall components that can take up heavy metal was reported elsewhere (Geddie and Sutherland 1993), and EPS have been documented to play a significant function in controlling heavy metal contamination in the sewage treatment processes (Kaewchai and Prasertsan 2002).

The EPS produced by most microorganisms reported in the literature is usually acidic polysaccharides with numerous carboxylate functional groups that carry negative charges that bind the metal ions (Ozdemir et al. 2003). The bioflocculant produced by *Bacillus firmus* could remove 98.3% of Pb^2+^, 74.9% of Cu^2+^, and 61.8% of Zn^2+^ from aqueous solution (Salehizadeh and Shojaosadati 2003). Rawat and Rai (2012) found that the bioflocculant produced by *Paenibacillus validus* MP5 demonstrated maximum adsorption values of 27%, 16%, 15%, 9%, and 7.5% for Zn^2+^, Ni^2+^ and Cd^2+^, Cr^2+^, Co^2+^, and Pb^2+^, respectively. The bioflocculant produced by *P. elgii* exhibited maximum adsorption activity for Al^3+^ at 72% with significant removal rates for Pb^2+^ (60%), Cu^2+^ (53%), and Co^2+^ (49%). Aluminum (Al^3+^) has a higher ionic valency thus increasing affinity to the EPS. Lin and Harichund (2012) reported that since bioflocculants have an extensive capacity for interacting with metals, they are recommended as surface‐active agents for the removal of heavy metals. A number of studies have also demonstrated the potential utilization of bioflocculants in heavy metal removal (Salehizadeh and Shojaosadati 2003; Wu and Ye 2007; Quintelas et al. 2008). Lin and Harichund (2011) reported the ability of bacterial bioflocculants in the removal of bacterial populations, heavy metals and turbidity from three industrial effluents.

The pollution caused by heavy metal deposition in aquatic systems is detrimental to both animal and human health and deserves special attention (Cristina et al. 2011). Therefore, the exploration of new technologies for the treatment of industrial wastewaters becomes essential. Gao et al. (2009) reported that the bioflocculant MBF4‐13 had a stronger removal efficiency of 69.3% for Cr_2_O_7_^2−^ than Ni^2+^ (19.2%). The bioflocculant MBF4‐13 mainly composed of polysaccharides that have hydroxyl groups in the molecular chain can easily form hydrogen bonds with Cr_2_O_7_^2−^, thereby resulting in higher removal efficiency than for Ni^2+^. Lin and Harichund (2011) found that the bioflocculant produced by *Paenibacillus* sp. CH11 had over 90% removal rate for Cd^2+^. Also, a novel glycoprotein bioflocculant MBF‐TG‐1 secreted by a strain of *Klebsiella* TG‐1 had a flocculation efficiency of about 86.9% for trona suspension. Several reported studies have shown that the bioflocculant produced by *Paenibacillus* had the ability to remove heavy metals from water (Morillo et al. 2006; Mokaddem et al. 2009; Rawat and Rai 2012).

## Decolorization of Dyeing Wastewater

The bioflocculant produced by *P*. *elgii* possesses functional groups that have the ability to decolorize cationic dyes in wastewater. This bioflocculant has a high removal rate of 65% for methylene blue and 72% for Red X‐GRL. Lower removal efficiencies that were below 50% were obtained when it was used to treat anionic and neutral dyes (Li et al. 2013). According to Li et al. (2013), there are two mechanisms of adsorption of cationic dyes by the bioflocculant. The acidic bioflocculant has a negatively charged COO^−^ group in the molecular chain which provides an adsorption site for the positively charged cationic dye molecules, thereby making electrostatic attraction possible (Verma et al. 2012). Furthermore, an analysis of the sugar content revealed that the bioflocculant possesses high levels of mannose which makes van der Waals force electrostatic interactions and hydrogen bonding possible (Blackburn 2004). Li et al. (2003) also documented a higher flocculating efficiency for REA‐11 secreted by *C. glutamicum* in comparison with chemically synthetic flocculants and found very effectual in the decolorization of molasses in wastewater. He et al. (2004) found that REA‐11 decolorizes molasses wastewater and proposed its potential for industrial application.

Bioflocculants have been applied in various industrial processes which included the flocculation of inorganic solid suspensions (Natarajan and Das 1999; Lu et al. 2005; Yim et al. 2007; Zhang et al. 2007). Dye removal in wastewater poses a serious challenge since largely all the dyes are absolutely soluble in aqueous solutions. The utilization of synthetic chemical dyes in different industrial processes such as the dyeing of cloth, paper, and pulp manufacturing, leather treatment, printing, and plastics has increased significantly recently, and this has led to the discharge of industrial effluents contaminated with dyes into the ecosystem (Aksu 2005). However, since some of these dyes are lethal in nature, their occurrence in industrial effluents will be a threat to the environment because they are usually not easily degraded by microorganisms (Pagga and Brown 1986).

In most cases, the partly degraded dyes from anaerobic degradation by some microorganisms generate potentially carcinogenic compounds that find their ways in the food chain and are later consumed by humans (Banat et al. 1996). Heavily colored wastewaters can block the access of sunlight and oxygen necessary for the survival of various aquatic forms (Crini 2006). The bioflocculant secreted by *Nannocystics* sp. Nu‐2 was recorded to be a glycoprotein which showed high efficiency in bleaching acid red and direct emerald blue (Zhang et al. 2002). Gao et al. (2009) reported that the bioflocculant MBF4‐13 produced by a novel bacterium strain ZHT4‐13 isolated from *R. philippinarum* conglutination mud was found to decolorize different dyes, with removal efficiencies of 86.11% for methylene blue, 97.84% for crystal violet, and 99.49% for malachite green. In addition, it was observed that this bioflocculant MBF4‐13 had a strong decolorizing efficiency for blue and violet series of dyes and possesses low decolorizing capability for red, pink, and orange series of dyes. Conversely, an observation by Deng et al. (2005) revealed that the bioflocculant secreted by *A. parasiticus* was more effective in removing Reactive Blue 4 and Acid Yellow 25 than Basic Blue B.

## Removal of Pathogens in Water

Oh et al. (2001) documented that the bioflocculant secreted by *Paenibacillus* sp. was effectively utilized in harvesting *Chlorella vulgaris* from a culture broth, while another bioflocculant produced by *P. polymyxa* AM49 was shown to be successful in the harvesting of a high density *Scenedesmus* sp. culture (Kim et al. 2011). Similarly, the bioflocculant produced from a culture broth of *S. silvestris* W01 demonstrated high flocculating activity of 90% on marine microalgae *Nannochloropsis oceanica* and as such has great prospects for harvesting marine microalgae for the commercial production of microalgal bioproducts. A similar report was observed for a bioflocculant produced by *S. silvestris* W01 (Wan et al. 2013). Also, another study by Zhao et al. (2013) suggests that the bioflocculant MBF‐5 produced by *K. pneumoniae* isolated from sputum samples showed a high flocculating rate of 84% in the removal of *Acanthamoeba* cysts, a potent pathogen in water and soil. The literature work on various bioflocculants and some of their industrial applications are summarized in Table 3.

**Table 3:** Literature work on bioflocculants and some of their applications

Application	Microorganism	Remarks	Reference
Removal of pathogens	*Klebsiella terrigena*	Remove *Salmonella* sp.	Ghosh et al. (2009)
	*Solibacillus silvestris* W01	Harvest *Nannochloropsis oceanica*	Wan et al. (2013)
	*Klebsiella pneumoniae*	Remove *Acanthamoeba* cysts	Zhao et al. (2013)
	*Paenibacillus polymyxa* AM49	Remove *Scenedesmus* sp.	Kim et al. (2011)
	*Bacillus agaradhaerens* C9	Harvest *Chlorella minutissima* UTEX2341	Liu et al. (2015)
Dye decolorization	*Ruditapes philippinarum*	Remove methylene blue, crystal violet, malachite	Wei et al. (2011)
	*Klebsiella* sp.	Remove sulfamethoxazole	Xing et al. (2013)
	*Serratia ficaria*	Decolorize pulp effluent	Gong et al. (2008)
	*Corynebacterium glutamicum*	Decolorize molasses wastewater	He et al. (2004)
	*Aspergillus parasiticus*	Decolorize Reactive Blue 4, Acid Yellow 25, Basic Blue B	Deng et al. (2005)
	*Staphylococcus* and *Pseudomonas* species	Decolorize indigotin printing and dyeing wastewater	Zhang et al. (2007)
	*Klebsiella mobilis*	Remove disperse yellow, disperse violet, reactive light yellow, and reactive turquoise blue	Wang et al. (2007a,b)
	*Paenibacillus polymyxa* BY‐28	Reactive brilliant blue and reactive brilliant yellow	Gong et al. (2003)
Water purification	*Paenibacillus elgii* B69	Real wastewater treatment	Li et al. (2013)
	*Bacillus mucilaginosus*	Treat domestic, brewery, and pharmaceutical wastewater	Lian et al. (2008)
	*Paenibacillus mucilaginosus* G1M16	Treat paper mill waterwater	Tang et al. (2014)
	*Bacillus licheniformis*	Treat sugar industry wastewater	Zhuang et al. (2012)
	*Oceanobacillus* and *Halobacillus* species	Treat brewery, dairy wastewater, and river water	Cosa and Okoh (2014)
	*Cobetia* and *Bacillus* species	Treat brewery, dairy wastewater, and river water	Ugbenyen and Okoh (2014)
	*Chlorella* sp. and *Micratinium* sp.	Industrial wastewater	Wang et al. (2014)
	*Arthrobacter* sp. B4	Treat alkaline wastewater	Li et al. (2014)
	*Azotobacter indicus*	Treat dairy, woollen, starch, and sugar wastewater	Patil et al. (2011)
	*Aspergillus niger*	Treat river water	Aljuboori et al. (2013)
	*Bacillus* sp.	Brewery waste water	Feng and Xu (2008)
Heavy metal removal	*Herbaspirillus* sp. CH7, *Paenibacillus* sp. CH 11, *Bacillus* sp. CH15, and *Halomonas* sp.	Pb^2+^, Zn^2+^, Hg^2+^, Cd^2+^	Lin and Harichund (2011)
	*Bacillus firmus*	Pb^2+^, Cu^2+^, and Zn^2+^	Salehizadeh and Shojaosadati (2003)
	*Paenibacillus validus* strain MP5	Zn^2+^, Ni^2+^, Cd^2+^, Cr^2+^, Co^2+^, and Pb^2+^	Rawat and Rai (2012)
	*Pseudomonas aeruginosa* strain IASST201	Ni^2+^, Co^2+^, Zn^2+^, Cu^2+^, Cd^2+^, Fe^2+^, Cr^2+^, and Mn^2+^	Pathak et al. (2014)
	*Achromobacter* sp.	Pb^2+^	Batta et al. (2013)
	*Klebsiella* sp. TG‐1	Defecating the trona suspension	Liu et al. (2013)
	*Enterobacter aerogenes*	Defecating the trona suspension	Lu et al. (2005)
	*Pseudomonas fluorescens* BM07	Hg^2+^, Cd^2+^, Ni^2+^, Zn^2+^, Cu^2+^, and Co^2+^	Noghabi et al. (2005)
	*Rhodococcus erythropolis*	Pb^2+^	Guo and Yu (2014)

## Molecular Biology and Synthesis of Bioflocculant

Genes involved in the synthesis of bioflocculants in different microbes are highly conserved and organized in clusters (Bai et al. 2008) and have been identified in some bioflocculant‐producing microbes such as in *Streptomyces* species, *B. licheniformis*, and *R. radiobacter* (Stingele et al. 1996; Tang et al. 1996; Li et al. 2003; Bai et al. 2008; Yan et al. 2013). The gene products, mostly enzymes, are involved in the formation of polysaccharides by sequential addition of sugars to membrane anchored repeating units which are then exported (Cerning 1990).

Biosynthetic processes can be controlled at three different levels: synthesis of sugar nucleotide precursors, assembly of the repeating unit, and polymerization and export (Bai et al. 2008). The modification of the expression of single genes or groups of genes can be used to increase the conversion efficiency of the chemical entities involved, and therefore, enhance bioflocculant yield. However, it might also provide a means of altering the polymer composition (Bajaj et al. 2007). Most bioflocculants are synthesized intracellularly and exported to the extracellular environment as macromolecules (Rehm 2009; Ullrich 2009).

Bacterial biosynthetic pathways comprise a substrate uptake, a central metabolite pathway, and a polysaccharide. Depending on the substrate type, it can be taken up by the cell either through a passive or an active transport system, following which it is catabolized by intracellular phosphorylation or it can be transported and oxidized through a direct oxidative periplasmic pathway. The periplasmic oxidative pathway exists only in certain bacteria, whereas the intracellular phosphorylative pathway is ubiquitous among bacteria. Both these systems have been reported in several bioflocculant‐producing microbes and they can function simultaneously if there is substrate availability (Schaechter and Lederberg 2004). In the cytoplasm, the substrate is catabolized through glycolysis and the primary metabolites formed are used as precursors for the synthesis of small biomolecules (e.g., amino acids or monosaccharides). Polysaccharide synthesis requires the biosynthesis of activated precursors that are energy‐rich monosaccharides, mainly nucleoside diphosphate sugars (NDP sugars), which are derived from phosphorylated sugars.

Even though bioflocculant production is a process that entails a perceptible energy cost, owing to the need for carbon as substrate for the growth of microorganisms, the gain together with their existence is significantly higher compared to the costs (taking into account the growth enhancement and the survival of the microbial producers) (Wolfaardt et al. 1999). As bioflocculant production is associated with a precise gene cluster, the information of the genome sequence of bioflocculant microbes is certainly the essential point for the optimization of their biosynthesis through the molecular biology approach (Ates et al. 2011, 2013). The production of bioflocculants is a genetically determined process and metabolic engineering is a powerful tool to improve metabolite productivity (Delbarre‐Ladrat et al. 2014).

Several gene clusters have been identified in both Gram‐positive and Gram‐negative bacteria that are involved in the biosynthesis of bioflocculant (Stingele et al. 1996). The enzymes encoded by these gene clusters can be divided into four groups: enzymes responsible for the initial metabolism of carbohydrates, enzymes involved in sugar nucleotide synthesis and interconversion, glycosyltranferases that form the repeating unit attached to the glycosyl carrier lipid, and translocases and polymerases that form the polymer (Looijesteijn et al. 1999). Therefore, in order to improve on the production, a precise approach is to identify all the genes responsible for bioflocculant synthesis and then attempt to understand the mechanisms involved. This is actually the research focus in biotechnology, paying attention to studies relating to the genomic level of bioflocculant‐producing microorganisms (Finore et al. 2014). Once the whole genome of these microorganisms has been sequenced, it will be suitable to select an appropriate tactic to improve the bioflocculant produced by manipulating those genes encoding the enzymes implicated in the bioflocculant synthesis (Yang et al. 2007). In addition, regulating the pathways that influence gene expression and enzyme activity, as well as the choice of the most appropriate substrate that will be supplemented with the media for cultivation of bioflocculant‐producing microbes ought to be considered (Yang et al. 2007). This could interfere with the physicochemical characteristics of the bioflocculants and may eventually have a great impact on bioflocculant properties and potential applications in industry. Nevertheless, the regulation of bioflocculant synthesis in marine microorganisms is still poorly understood (Bajaj et al. 2007; Rehm 2009) and it will be vital to explore the advances in genetic engineering of bioflocculant‐producing microbes in order to improve yields.

## Conclusion and Future Prospects

Chemical flocculants are effective at aggregating colloids and have been widely used in different industrial processes. Because of their negative health impacts and the environmental hazards associated with chemical flocculants, microbial flocculants have gained huge scientific and biotechnology consideration because of their safety and ecofriendly attributes.

Marine habitats which support a rich biodiversity of marine bacteria remain underexplored for this purpose and yet hold tremendous promise as reservoirs of novel bioflocculant‐producing organisms. Although many bioflocculants have been reported in the literature, their large‐scale production is still limited by low yields, high production costs, and low flocculating activity. Optimization of media constituents and fermentation conditions is also one of the strategies to improve on bioflocculants yields and flocculating activity. However, the high cost of media constituents, will make its highly propitious to utilize cost‐effective substrates for large‐scale bioflocculant production in industries. Furthermore, the utilization of microorganisms in consortia for bioflocculants production that will possess better flocculating activity and higher bioflocculant yield than pure strains is essential. Furthermore, additional knowledge of the genetics and biochemistry of bioflocculant biosynthesis is imperative before their production processes are modified for better yield and increased activities which are subjects of ongoing investigations in our group.

The future development of microbial flocculants will depend on a number of factors, but the key question is whether they can be produced economically. Utilization of agricultural wastes or industrial wastewaters (possibly along with other substrates) is certainly a possibility for bioflocculant production. Only a limited number of microbial species show diverse enough substrate utilization for agricultural wastes to be suitable substrates. Considerable research will be necessary to ensure that bioflocculant synthesized using agricultural waste substrates are of satisfactory quality and have acceptable properties. This will reasonably cut down production cost and encourage their large‐scale production and industrial application.

In addition, only a few cation‐independent bioflocculants have been identified and documented in the literature. Therefore, further studies are needed to produce cation‐independent bioflocculants with high flocculating efficiencies and consequently reduce the environmental pollution caused by the cations used in the flocculation processes.

The flocculation optimization practices in the industry are still scarce because of the highly complex nature of the flocculation process and the large variety of polyelectrolytes available. One of the ways to optimize the flocculation process is by selecting or controlling the range of the molecular weight and the charge density of the bioflocculant. Different molecular weights and charge densities produce different flocculation mechanisms (neutralization or bridging). Future research needs to look into how molecular weight and charge density distribution affect the flocculation performance to produce a better choice of bioflocculants for specific industrial applications. Optimization of these factors could significantly increase the treatment efficiency and reduce the chemical cost.

In addition, very limited work has been carried out on the industrial scale. Most reports have concentrated on laboratory studies. The complexity of the coagulation and flocculation systems justifies that a bioflocculant cannot be selected for a given application without experimental testing. Industrial trials or practices for confirming the dosage suitable and other physicochemical conditions for flocculation are still lacking. Furthermore, the applicability and effectiveness of these bioflocculants for wastewater treatment in large scale is yet to be established. Further investigation on the industrial scale‐up conditions is highly imperative.

The selection of highly efficient bioflocculants that can remove all contaminants in wastewater is essential for a successful flocculation process. Environmental friendly bioflocculants that can be produced by simple and economically viable process which exhibits high removal efficiencies and considerably denser flocs is regarded as a promising material for application from the perspective of both performance and cost. In order to control and optimize the flocculation process, it is very important to understand the flocculation mechanism during the whole process. However, the investigation and discovery of the underlying mechanism for removal of impurities or contaminants from wastewater with bioflocculants is still lacking and immature and so requires attention.

Development of suitable bioflocculant extraction methods is one of the factors that affect the property of the purified product. Suitable extraction methods with a high efficiency should be pursued. Such methods should be mild to avoid the lysis of cells and the disruption of bioflocculant characteristics.

Most bioflocculant‐producing microorganisms are usually incubated at or near 30°C, although incubation at suboptimal temperatures conventionally favors bioflocculant production. There would be obvious advantages in using thermophiles capable of growth at higher temperatures in order to avoid the necessity of expensive cooling systems for large‐scale synthesis in the industries. Nevertheless, none of these bacteria have yet proved to be sources of bioflocculant with good rheological properties. Further research will be crucial to isolate these thermophile microorganisms from different environments that will be utilized for bioflocculant production.

Finally, it will be crucial to establish an appropriate fermentation (fed‐batch vs. continuous fermentation) conditions for scale‐up process for bioflocculant production. Furthermore, to determine the shelf life of the bioflocculant as well as establish appropriate packaging regimes. It will be imperative to carry out feasibility study on the marketability of the final bioflocculant product. For the bioflocculant to be of industrial benefit, all the aforementioned points must be put into consideration.

## Conflict of Interest

None declared.
